# Risk of Chronic Cardiomyopathy Among Patients With the Acute Phase or Indeterminate Form of Chagas Disease: A Systematic Review and Meta-analysis

**DOI:** 10.1001/jamanetworkopen.2020.15072

**Published:** 2020-08-31

**Authors:** Sindhu Chadalawada, Stefan Sillau, Solana Archuleta, William Mundo, Mehdi Bandali, Gabriel Parra-Henao, Alfonso J. Rodriguez-Morales, Wilmer E. Villamil-Gomez, José Antonio Suárez, Leland Shapiro, Peter J. Hotez, Laila Woc-Colburn, Kristen DeSanto, Anis Rassi, Carlos Franco-Paredes, Andrés F. Henao-Martínez

**Affiliations:** 1 NRI Medical College, Guntur, Andhra Pradesh, India; 2 Department of Neurology, University of Colorado Denver, Denver; 3 University of Colorado School of Medicine, Aurora; 4 National Institute of Health, Bogotá, Colombia; 5 Centro de Investigación en Salud para el Trópico (CIST), Universidad Cooperativa de Colombia, Santa Marta, Colombia; 6 Public Health and Infection Research Group, Faculty of Health Sciences, Universidad Tecnológica de Pereira, Pereira, Risaralda, Colombia; 7 Grupo de Investigación Biomedicina, Faculty of Medicine, Fundación Universitaria Autónoma de las Américas, Pereira, Risaralda, Colombia; 8 Departamento de Infectología, Hospital Universitario de Sincelejo, Sucre, Colombia; 9 Investigador Sistema Nacional de Investigación, Secretaría Nacional de Ciencia, Tecnología e Innovación, Clinical Research Department, Instituto Conmemorativo Gorgas de Estudios de la Salud, Panamá City, Panamá; 10 Rocky Mountain Regional Veterans Affairs Medical Center, Aurora, Colorado; 11 Division of Infectious Diseases, Department of Medicine, University of Colorado Denver, Denver; 12 Texas Children’s Hospital Center for Vaccine Development, Texas Children’s Hospital, Houston, Texas; 13 Department of Pediatrics, Baylor College of Medicine, Houston, Texas; 14 Department of Molecular Virology and Microbiology, National School of Tropical Medicine, Baylor College of Medicine, Houston, Texas; 15 Division of Infectious Diseases, Emory University School of Medicine, Atlanta, Georgia; 16 Health Sciences Library, University of Colorado Denver, Aurora; 17 Division of Cardiology, Anis Rassi Hospital, Goiania, Goias, Brazil; 18 Hospital Infantil de México Federico Gómez, Mexico City, Mexico

## Abstract

**Importance:**

Chagas cardiomyopathy is associated with substantial morbidity and mortality. Precise estimates of the risk of developing cardiomyopathy among patients with the acute or indeterminate chronic forms of Chagas disease are lacking.

**Objective:**

To estimate the risk of developing chronic cardiomyopathy in patients with acute and indeterminate chronic forms of Chagas disease.

**Data Sources:**

A systematic search in the Cochrane Library, Embase, Latin American and Caribbean Health Sciences Literature (LILACS), Medline, and Web of Science Core Collection databases was conducted from October 8 to October 24, 2018. Studies published between January 1, 1946, and October 24, 2018, that were written in the English, Spanish, and Portuguese languages were included. Search terms included *Chagas disease*; *development of cardiomyopathy*; *latency duration*; and *determinants of the Chagas latency period*.

**Study Selection:**

Longitudinal observational studies of participants diagnosed with the acute phase of Chagas infection or the indeterminate chronic form of Chagas disease who were followed up until the development of cardiomyopathy were included. Studies were excluded if they did not provide sufficient outcome data. Of 10 761 records initially screened, 32 studies met the criteria for analysis.

**Data Extraction and Synthesis:**

Critical appraisals of studies were performed using checklists from the *Joanna Briggs Institute Reviewer’s Manual*, and data were collected from published studies. A random-effects meta-analysis was used to obtain pooled estimated annual rates. Data were analyzed from September 11 to December 4, 2019. This study followed the Preferred Reporting Items for Systematic Reviews and Meta-analyses (PRISMA) reporting guideline for the registration of the protocol, data collection and integrity, assessment of bias, and sensitivity analyses.

**Main Outcomes and Measures:**

Main outcomes were defined as the composite of the development of any new arrhythmias or changes in electrocardiogram results, dilated cardiomyopathy and segmental wall motion abnormalities in echocardiogram results, and mortality associated with Chagas disease.

**Results:**

A total of 5005 records were screened for eligibility. Of those, 298 full-text articles were reviewed, and 178 of those articles were considered for inclusion in the quantitative synthesis. After exclusions, 32 studies that included longitudinal observational outcomes were selected for the analysis; 23 of those studies comprised patients with the indeterminate chronic form of Chagas disease, and 9 of those studies comprised patients in the acute phase of Chagas infection. The analysis indicated that the pooled estimated annual rate of cardiomyopathy development was 1.9% (95% CI, 1.3%-3.0%; *I^2^* = 98.0%; τ^2^ [ln scale] = 0.9992) in patients with indeterminate chronic Chagas disease and 4.6% (95% CI, 2.7%-7.9%; *I^2^* = 86.6%; τ^2^ [ln scale] = 0.4946) in patients with acute Chagas infection.

**Conclusions and Relevance:**

Patients with the indeterminate chronic form of Chagas disease had a significant annual risk of developing cardiomyopathy. The annual risk was more than double among patients in the acute phase of Chagas infection.

## Key Points

**Question:**

What is the risk of developing cardiomyopathy among patients with the acute phase of Chagas infection or the indeterminate chronic form of Chagas disease?

**Findings:**

In this systematic review and meta-analysis of 32 studies of patients with Chagas disease, the pooled estimated annual rate of cardiomyopathy was 4.6% among patients with acute Chagas infection and 1.9% among patients with indeterminate chronic Chagas disease.

**Meaning:**

The findings indicate that asymptomatic individuals with indeterminate chronic Chagas disease without cardiac injury and individuals with acute Chagas infection may have a significant risk of developing chronic cardiomyopathy.

## Teaser

This systematic review and meta-analysis assesses the risk of developing chronic cardiomyopathy among patients with acute and indeterminate chronic forms of Chagas disease.

## Introduction

Approximately 6 million people in Latin America have Chagas disease, and more than 70 million people are at risk of developing the infection.^1^ The hemoflagellate protozoan *Trypanosoma cruzi* is the infectious agent. Transmission is most commonly vectorial through a bite from the triatomine insect (commonly known as the kissing bug). Other important routes of *T cruzi* transmission are oral (through food or drink contaminated with triatomine feces or secretions from infected mammals), vertical (or congenital, from mother to child during pregnancy), organ transplantation, blood transfusion, and unintentional laboratory exposure.

Chagas disease has 2 phases, acute and chronic. In the acute phase, which occurs during the first few weeks or months after infection, the disease is often subclinical and undiagnosed, as patients are asymptomatic or present with nonspecific symptoms, such as fever, malaise, hepatosplenomegaly, skin edema (termed chagoma, an inflammatory nodule at the site of the triatomine bite), or edema of 1 or both eyelids (termed the Romaña sign, a sensitization response to the triatomine bite). However, cardiac involvement may also occur and is generally indicative of a worse prognosis.^2^ The chronic phase of Chagas disease has 2 forms: indeterminate and determinate. The indeterminate form occurs after the acute phase and may last for decades (or even a lifetime) without symptoms. The indeterminate form can progress to the determinate form, which includes the development of cardiomyopathy, digestive disease, or cardiodigestive disease.^3^ The primary factor associated with morbidity in patients with Chagas disease is the development of chronic Chagas cardiomyopathy, which is manifested in heart failure, systemic and pulmonary embolism, arrhythmia, and sudden cardiac death. Diagnosis and treatment of patients with acute Chagas infection or the indeterminate chronic form of Chagas disease may decrease the risk of cardiomyopathy, vertical transmission, and death.^4,5,6,7^

The Chagas latency period encompasses the asymptomatic period from the end of the acute phase until the development of a determinate form of the disease. Early studies and clinical observations have estimated the latency period to be approximately 10 to 30 years. A longitudinal study in Brazil conducted from 1974 to 1984 found a progression rate of 38% from the indeterminate chronic form to the development of chagasic cardiomyopathy over the 10-year follow-up period.^8^ A cohort study in Argentina found a more rapid progression rate of 21% in less than 3 years.^9^ Oral transmission is also associated with a higher incidence of myocarditis and mortality.^10^

The risk of developing cardiomyopathy in patients with latent Chagas disease has prompted clinicians and public health agencies to design strategies to optimize Chagas screening programs and improve the treatment of asymptomatic individuals. However, as reported in several longitudinal studies, data regarding the risk of cardiomyopathy and its factors are inconsistent. A more informative and detailed analysis of contemporary data can be important to achieving a better understanding of the natural progression of Chagas disease. Through a systematic review and meta-analysis, we aimed to estimate the progression rates to cardiomyopathy among patients with the acute or the indeterminate chronic forms of Chagas disease.

## Methods

### Search Strategy and Selection Criteria

We performed a systematic review and meta-analysis of studies of cardiomyopathy development in patients with Chagas disease. The systematic review considered studies that explored the rate of progression to cardiomyopathy among patients with acute or indeterminate chronic Chagas disease. Longitudinal observational studies of participants diagnosed with the acute phase of Chagas infection or the indeterminate chronic form of Chagas disease who were followed up until the development of cardiomyopathy were included. Studies were excluded if they did not include sufficient outcome data. The detailed methods and Medline search strategy are available in eMethods in the Supplement. Additional details on the systematic review protocol have been published previously (PROSPERO CRD42019118019).^11^ This study followed the Preferred Reporting Items for Systematic Reviews and Meta-analyses (PRISMA) reporting guideline for the registration of the protocol, data collection and integrity, assessment of bias, and sensitivity analyses.

A systematic search in the Cochrane Library, Embase, Latin American and Caribbean Health Sciences Literature (LILACS), Medline, and Web of Science Core Collection databases was conducted from October 8 to October 24, 2018. Studies published between January 1, 1946, and October 24, 2018, that were written in the English, Spanish, and Portuguese languages were included. Search terms included *Chagas disease*; *development of cardiomyopathy*; *latency duration*; and *determinants of the Chagas latency period*.

### Data Analysis and Quality Assessment

Study data were collected and managed using REDCap electronic data capture tools hosted at the University of Colorado Denver. Extracted data included the type of study, the study’s country of origin, the number of participants, the duration of follow-up, the demographic characteristics of the study population, and the study’s methods, interventions (diagnostic and therapeutic), and primary outcomes of interest. The following primary cardiac outcomes were extracted: (1) the development of cardiac or heart failure symptoms, including shortness of breath, dyspnea on exertion, lower extremity edema, paroxysmal nocturnal dyspnea, and orthopnea; (2) the development of structural cardiomyopathy or cardiac arrhythmias, as observed in abnormal echocardiogram and/or abnormal electrocardiogram (ECG) results; and (3) the presence of complications associated with advanced cardiomyopathy, including mortality associated with advanced heart failure, sudden death, pulmonary embolism, or stroke. Secondary outcomes, such as hospitalization rates, heart transplant receipt status, and implantable cardioverter-defibrillator or pacemaker requirements, which were outlined in the initial protocol, were not analyzed owing to insufficient data.

Two reviewers with expertise in Chagas disease (A.H.M. and C.F.P.) independently reviewed the selected studies for methodological quality and performed quality standard measures using the System for the Unified Management, Assessment and Review of Information (SUMARI; Joanna Briggs Institute) software. A third reviewer (S.C.) was asked to reconcile any disagreements between the 2 reviewers. Critical appraisals were performed using checklists from the *Joanna Briggs Institute Reviewer’s Manual* for cohort studies, case-control studies, case-series studies, case reports, and cross-sectional studies.^12^ All studies that had positive answers of more than 60% for the critical appraisal questions were subject to data extraction and synthesis.

### Statistical Analysis

Cardiac events were defined as the composite of the development of any new arrhythmias or changes in ECG results, dilated cardiomyopathy or segmental wall motion abnormalities in echocardiogram, and mortality associated with Chagas disease. Event rates were extracted by calculating the cumulative percentage of cardiac events among the participants over the study duration, assuming a constant exponential hazard time to event distribution and solving for the rate. The extracted ratio was log transformed, and SEs were obtained from the cumulative percentage of cardiac events, the number of participants, and the duration of the study using the delta method. Because the studies were conducted over varying lengths of time, and we could only obtain the proportion of events that occurred at the studies’ end points, a constant rate had to be assumed. Log transformation of the estimated rates reduced the skew of their distributions.

A random-effects meta-analysis was then performed to combine the estimated log rates from the different studies into a single estimated log rate, which was back-transformed for interpretation. Between-study heterogeneity was estimated using the *I*^2^ statistic. Subgroup and meta-regression analyses were conducted to examine other factors of cardiomyopathy development and sources of heterogeneity; these analyses included the study’s country of origin, the participants’ ages, and the percentages of men vs women. A post hoc analysis was performed to examine antiparasitic treatments received (per study arm), year of study, study size and duration, and route of disease transmission. Continuous variables were dichotomized using the mean. When high heterogeneity was obtained, we performed a subgroup analysis or removed selected studies to assess the source of the high heterogeneity. Contour-enhanced funnel plots were constructed to assess publication bias. Statistical analyses were performed using Stata software, version 16.0 (StataCorp). Data were analyzed from September 11 to December 4, 2019.

## Results

**Figure 1.**
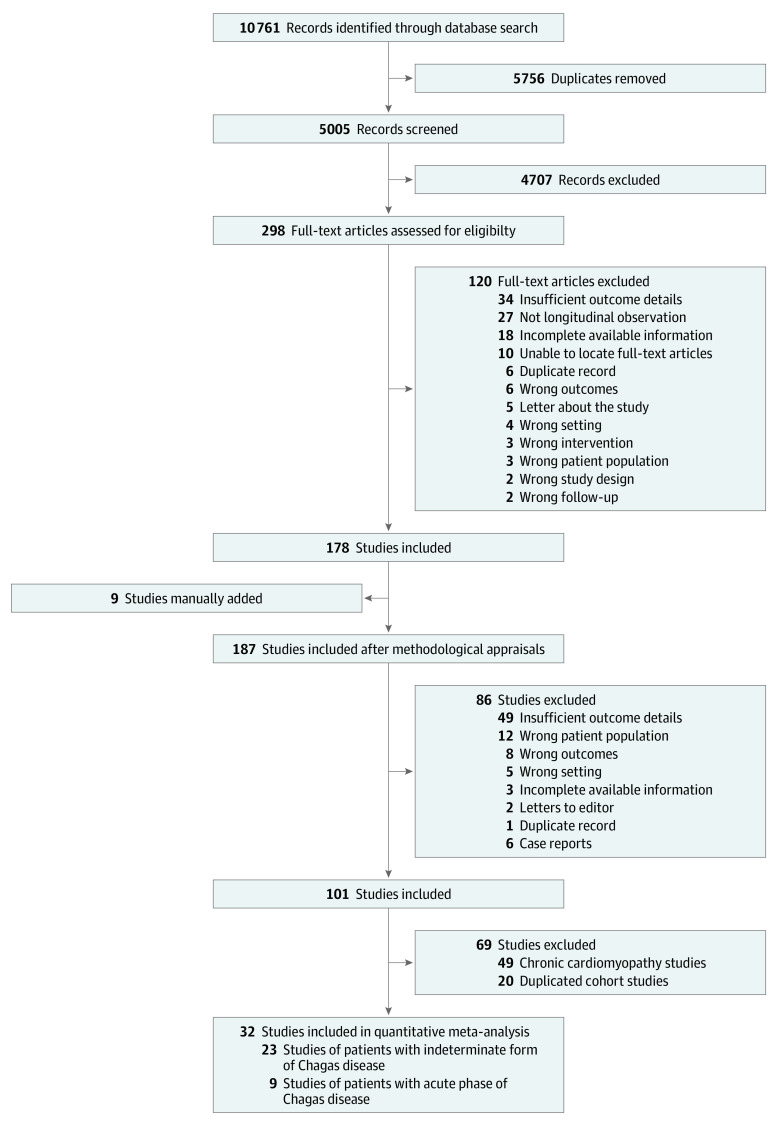
PRISMA Flow Diagram

A total of 10 761 records were identified through database searches. Of those, 5005 records were screened for eligibility based on titles and abstracts; 298 full-text articles were reviewed, and 178 of those articles were considered for inclusion in the quantitative synthesis (Figure 1). Nine articles were then manually added, for a total of 187 studies. Of those, 101 studies passed the initial methodological appraisal. We then excluded 20 studies because they originated from identical cohorts and 49 studies because they included patients who had already received a diagnosis of chronic cardiomyopathy. After all exclusions, 32 studies were included in the meta-analysis, 23 of which comprised patients with the indeterminate chronic form of Chagas disease and 9 of which comprised patients with acute Chagas infection at the onset of observation.

### Indeterminate Chronic Chagas Infection

**Figure 2.**
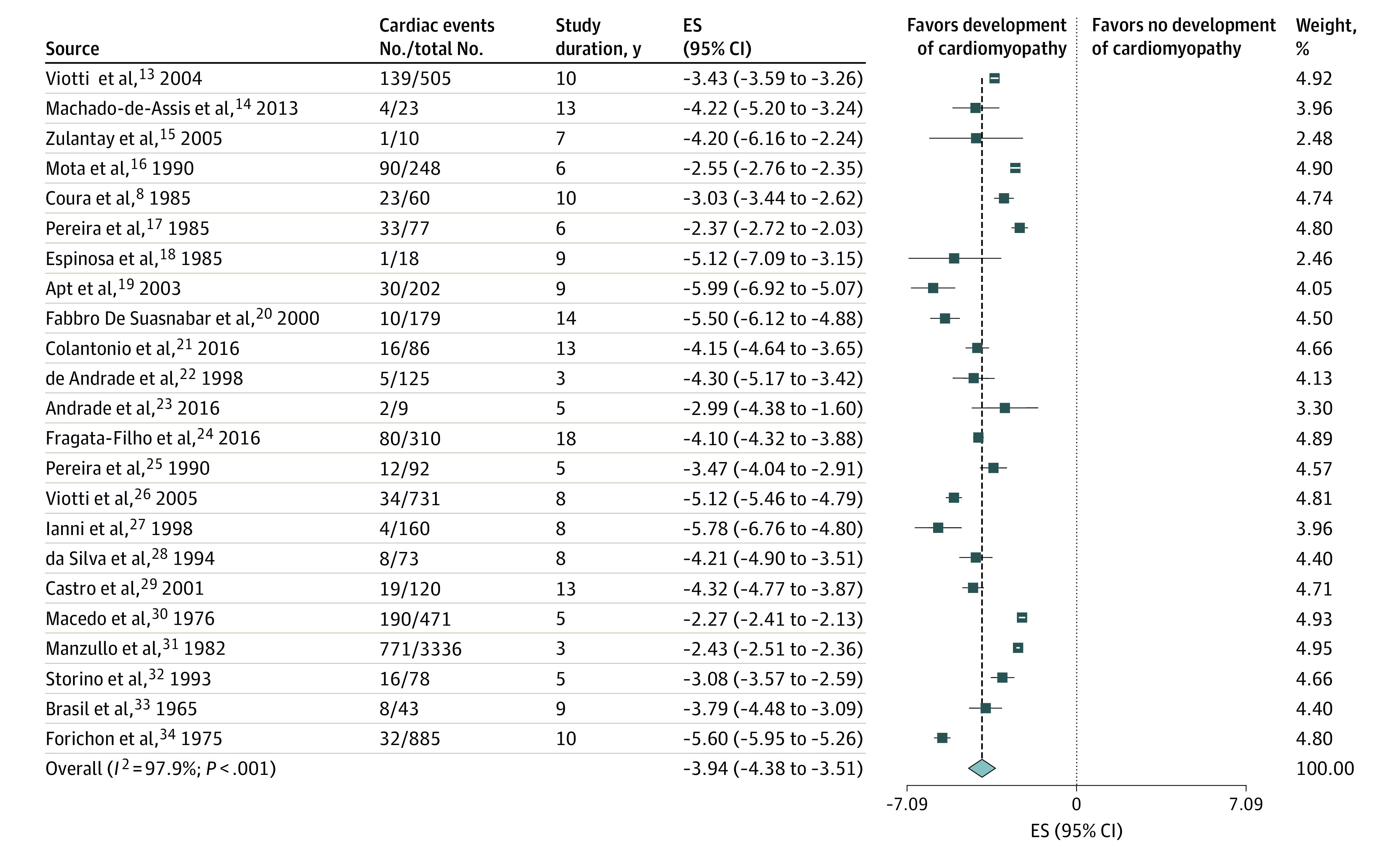
Forest Plot of Cardiomyopathy Risk in Studies of Patients With the Indeterminate Chronic Form of Chagas Disease A greater negative logarithmic estimated rate converts to a lower back-transformed rate. Weights are from random-effects analysis. ES indicates effect size.

Twenty-three studies^8,13,14,15,16,17,18,19,20,21,22,23,24,25,26,27,28,29,30,31,32,33,34^ had longitudinal observational outcomes for patients with the indeterminate chronic form of Chagas disease (Table 1). Most of these studies were of prospective cohorts and were conducted in either Brazil or Argentina between 1960 and 2005. Samples ranged from 9 to 3336 participants, with a mean of 345 participants per study. Distribution among sexes was equal, with a mean of 46% men per study. Not all studies had age data available. Among studies that included age data, the ages ranged from 10 to 44 years, with a mean age of 31 years. The mean follow-up duration was 8.5 years, with a range of 3 to 18 years. The pooled estimated annual rate of the development of chronic Chagas cardiomyopathy was 1.9% (95% CI, 1.3%-3.0%; *I^2^* = 98.0%; τ^2^ [ln scale] = 0.9992) (Figure 2). The cumulative probability of experiencing a cardiac event was approximately 17% at 10 years and 31% at 20 years (eFigure 1 in the Supplement).

**Table 1.** Baseline Characteristics and Clinical Outcomes of Patients With the Indeterminate Chronic Form of Chagas Disease

Source	Study design	Country	Participants, No.	Male sex, No. (%)	Age, mean, y	Intervention^a^	Study duration, y	Cardiac events, No. (%)^b^	Estimated rate (95% CI)^c^	Weight, %
Viotti et al,^13^ 2004	Cross-sectional	Argentina	505	333 (65.9)	40.5	None	9.9	139 (27.5)	3.3 (2.8-3.8)	4.9
Machado-de-Assis et al,^14^ 2013	Prospective cohort	Brazil	23	8 (34.8)	26.7	Benznidazole therapy	13.0	4 (17.4)	1.5 (0.6-3.9)	4.0
Zulantay et al,^15^ 2005	Prospective cohort	Chile	10	NA	NA	Other antiparasitic therapy^d^	7.0	1 (10.0)	1.5 (0.2-10.7)	2.5
Mota et al,^16^ 1990	Prospective cohort	Brazil	248	108 (43.5)	NA	None	5.8	90 (36.3)	7.8 (6.3-9.6)	4.9
Coura et al,^8^ 1985	Prospective cohort	Brazil	60	NA	NA	None	10.0	23 (38.3)	4.8 (3.2-7.3)	4.7
Pereira et al,^17^ 1985	Case-control	Brazil	77	NA	31.2	None	6.0	33 (42.9)	9.3 (6.6-13.2)	4.8
Espinosa et al,^18^ 1985	Prospective cohort	Venezuela	18	9 (50.0)	37.0	None	9.4	1 (5.6)	0.6 (0.1-4.3)	2.5
Apt et al,^19^ 2003	Randomized clinical trial	Chile	202	NA	NA	Other antiparasitic therapy^d^	9.0	30 (14.9)	0.2 (0.1-0.6)	4.1
Fabbro De Suasnabar et al,^20^ 2000	Prospective cohort	Argentina	179	NA	NA	Benznidazole or nifurtimox therapy^e^	14.0	10 (5.6)	0.4 (0.2-0.8)	4.5
Colantonio et al,^21^ 2016	Retrospective cohort	Argentina	86	NA	10.0	Benznidazole therapy or placebo^f^	13.0	16 (18.6)	1.6 (1.0-2.6)	4.7
de Andrade et al,^22^ 1998	Prospective cohort	Brazil	125	NA	10.4	None	3.0	5 (4.0)	1.4 (0.6-3.3)	4.1
Andrade et al,^23^ 2016	Prospective cohort	Brazil	9	NA	NA	Benznidazole therapy	5.0	2 (22.2)	5.0 (1.3-20.2)	3.3
Fragata-Filho et al,^24^ 2016	Prospective cohort	Brazil	310	107 (34.5)	34.5	Benznidazole therapy^g^	18.0	80 (25.8)	1.7 (1.3-2.1)	4.9
Pereira et al,^25^ 1990	Prospective cohort	Brazil	92	NA	39.6	None	4.5	12 (13.0)	3.1 (1.8-5.5)	4.6
Viotti et al,^26^ 2005	Prospective cohort	Argentina	731	355 (48.6)	43.7	None	8.0	34 (4.7)	0.6 (0.4-0.8)	4.8
Ianni et al,^27^ 1998	Prospective cohort	Brazil	160	62 (38.8)	36.5	None	8.2	4 (2.5)	0.3 (0.1-0.8)	4.0
da Silva et al,^28^ 1994	Prospective cohort	Brazil	73	NA	NA	None	7.8	8 (11.0)	1.5 (0.7-3.0)	4.4
Castro et al,^29^ 2001	Prospective cohort	Brazil	120	NA	NA	None	13.0	19 (15.8)	1.3 (0.8-2.1)	4.7
Macedo,^30^ 1976	Prospective cohort	Brazil	471	NA	NA	None	5.0	190 (40.3)	10.3 (8.9-11.9)	4.9
Manzullo et al,^31^ 1982	Prospective cohort	Multicenter^h^	3336	1944 (58.3)	NA	None	3.0	771 (23.1)	8.8 (8.2-9.4)	5.0
Storino,^32^ 1993	Prospective cohort	Argentina	78	35 (44.9)	36.1	None	5.0	16 (20.5)	4.6 (2.8-7.5)	4.7
Brasil,^33^ 1965	Prospective cohort	Brazil	43	NA	NA	None	9.1	8 (18.6)	2.3 (1.1-4.5)	4.4
Forichon,^34^ 1975	Prospective cohort	Brazil	885	373 (42.1)	NA	None	10.0	32 (3.6)	0.4 (0.3-0.5)	4.8

Abbreviation: NA, not available or not applicable.

^a^ Antiparasitic treatment.

^b^ Cardiac events include the development of any new changes in electrocardiogram results, arrhythmias, dilated cardiomyopathy, segmental motion abnormality, or mortality associated with Chagas disease.

^c^ Estimated rate was calculated using the exponential survival method (1 divided by the number of years multiplied by the − logarithmic function of [100 minus the number of cardiac events divided by the total number of participants]).

^d^ Allopurinol and itraconazole therapy.

^e^ Two cardiac events occurred in 63 participants in the treatment arm, and 8 cardiac events occurred in 116 participants in the control arm.

^f^ Eight cardiac events occurred in 48 participants in the treatment arm, and 8 cardiac events occurred in 38 participants in the control arm.

^g^ A total of 55 cardiac events occurred in 263 participants in the treatment arm, and 25 cardiac events occurred in 47 participants in the control arm.

^h^ Centers included Argentina, Bolivia, Brazil, Chile, Paraguay, Uruguay, and Peru.

Subgroup and post hoc analyses indicated no difference between studies based on year of study (2.8% for studies before 1985 vs 1.4% for studies after 1985; *P* = .10), study size (1.9% for studies with <200 participants vs 2.1% for studies with >200 participants; *P* = .83), mean age of participants (2.4% for studies with a mean participant age of <32 years vs 1.6% for studies with a mean participant age of >32 years; *P* = .84), and percentage of men (1.0% for studies comprising <40% men vs 2.2% for studies comprising >40% men; *P* = .46) (eTable 1, eFigure 3, eFigure 4, eFigure 5, and eFigure 6 in the Supplement). Among studies originating in Brazil, participants had a significantly higher annual rate of cardiomyopathy development (2.3%; 95% CI, 1.2%-4.3%) compared with studies of patients from other South American countries (1.1%; 95% CI, 0.5%-2.4%; *P* = .05; 1 study was omitted because it was conducted in both Brazil and Peru) (eTable 1 and eFigure 7 in the Supplement).

The post hoc subgroup of participants who received antiparasitic treatment had a significantly lower pooled estimated annual rate of cardiomyopathy development (1.0%; 95% CI, 0.5%-1.9%) compared with the subgroup who did not receive treatment (2.3%; 95% CI, 1.5%-3.5%; *P* = .03) (eTable 1 and eFigure 8 in the Supplement). Studies of longer duration (≥9 years) also had a significantly lower pooled estimated annual rate than those of shorter duration (<9 years; 1.2% vs 3.2%, respectively; *P* = .001) (eTable 1 and eFigure 9 in the Supplement). Heterogeneity remained high after we individually removed specific studies; however, the pooled estimated rates were consistent at a range of 1.6% to 1.9%.

### Acute Chagas Infection

**Figure 3.**
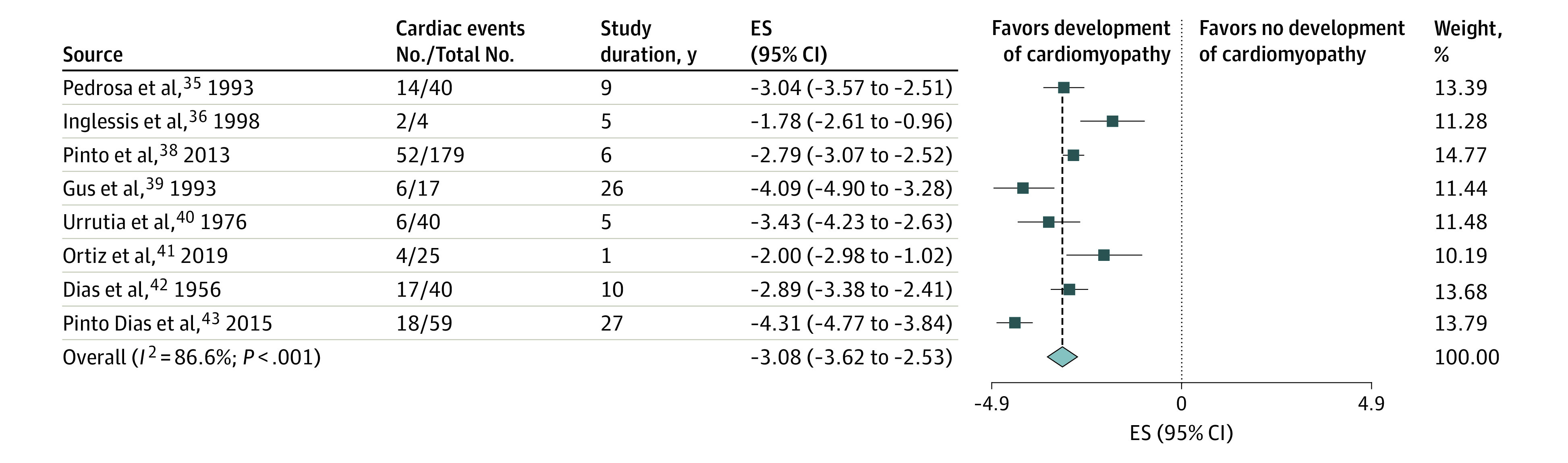
Forest Plot of Cardiomyopathy Risk in Studies of Patients With the Acute Form of Chagas Disease A greater negative logarithmic estimated rate converts to a lower back-transformed rate. Weights are from random-effects analysis. ES indicates effect size.

Nine studies^35,36,37,38,39,40,41,42,43^ included longitudinal observational outcomes for patients with acute Chagas infection (Table 2). These studies were primarily case series or prospective cohorts performed between 1940 and 2006 in Brazil, Venezuela, and El Salvador. The main routes of transmission in these studies were vectorial followed by oral. The mean number of participants per study was 50, with an even distribution of men and women and a mean age of 26 years. The duration of longitudinal observation ranged from 6 months to 27 years. The pooled estimated rate of the development of chronic Chagas cardiomyopathy was 6.8% (95% CI, 3.3%-14.2%; *I^2^* = 93.3%; τ^2^ [ln scale] = 1.1347) (eFigure 10 in the Supplement). The cumulative probability of a cardiac event was approximately 49% at 10 years. After removal of 1 study that had a follow-up period of only 6 months,^37^ we found a pooled estimated annual rate of 4.6% (95% CI, 2.7%-7.9% per year; *I^2^* = 86.6%; τ^2^ [ln scale] = 0.4946), with a modest decrease in heterogeneity (Figure 3), and a cumulative probability of experiencing a cardiac event of approximately 40% at 10 years (eFigure 2 in the Supplement).

**Table 2.** Baseline Characteristics and Clinical Outcomes of Patients With the Acute Form of Chagas Disease

Source	Study design	Country	Type of transmission	Participants, No.	Male sex, No. (%)	Age, mean, y	Study duration, y	Cardiac events, No. (%)^a^	Estimated rate (95% CI)^b^	Weight, %
Pedrosa et al,^35^ 1993	Case series	Brazil	Vectorial	40	24 (60.0)	NA	9.0	14 (35.0)	4.8 (2.8-8.1)	11.6
Inglessis,^36^1998	Case series	Venezuela	Vectorial	10	6 (60.0)	23.0	5.5	6 (60.0)	16.8 (7.3-38.4)	10.7
Bastos et al,^37^ 2010	Case series	Brazil	Oral	11	8 (72.7)	24.6	0.5	6 (54.5)	157.5 (69.1-359.3)	10.7
Pinto et al,^38^ 2013	Prospective cohort	Brazil	Oral	179	NA	NA	5.5	52 (29.1)	6.1 (4.7-8.1)	12.1
Gus et al,^39^ 1993	Case series	Brazil	Oral	17	8 (47.1)	30.4	26.0	6 (35.3)	1.7 (0.7-3.8)	10.7
Urrutia,^40^ 1976	Case series	El Salvador	Vectorial	40	14 (35.0)	NA	5.0	6 (15.0)	3.3 (1.5-7.2)	10.7
Ortiz et al,^41^ 2019	Prospective cohort	Brazil	Oral	25	NA	NA	1.3	4 (16.0)	13.5 (5.1-36.1)	10.1
Dias et al,^42^ 1956	Prospective cohort	Brazil	Vectorial	40	NA	NA	10.0	17 (42.5)	5.5 (3.4-9.0)	11.7
Pinto Dias,^43^ 2015	Prospective cohort	Brazil	Vectorial	59	NA	NA	27.0	18 (30.5)	1.3 (0.8-2.1)	11.7

Abbreviation: NA, not available or not applicable.

^a^ Cardiac events include the development of any new arrhythmias, changes in electrocardiogram results, or sudden death.

^b^ Estimated rate was calculated using the exponential survival method (1 divided by the number of years multiplied by the − logarithmic function of [100 minus the number of cardiac events divided by the total number of participants]).

After exclusion of the study with a follow-up period of 6 months, the subgroup post hoc analysis based on the year of study onset (before or after 1975, with 1975 representing the mean of all years of study onset) indicated statistically higher pooled estimated annual rates of cardiomyopathy development for studies after 1975 compared with those before 1975 (10.1% vs 2.9%, respectively; *P* = .007) (eTable 2 and eFigure 11 in the Supplement). A meta-regression analysis with an indicator variable revealed an estimated rate ratio for pre-1975 studies compared with post-1975 studies of 3.49 (95% CI, 1.09–11.19; *P* = .04; *I*^2^ = 79.7; τ^2^ [ln scale] = 0.3094), which represented an increase of 249%. By modeling the year of study onset as a continuous variable in the meta-regression analysis, we estimated a statistically significant rate increase of 3.32% per year (estimated rate ratio, 1.03; 95% CI, 1.01-1.06; *P* = .02; *I*^2^ = 68.2; τ^2^ [ln scale] = 0.1922).

In our subgroup and post hoc analyses, the results were not substantially changed by the study’s size (7.2% for studies with <40 participants vs 3.8% for studies with >40 participants; *P* = .42), the study’s country of origin (4.0% for studies performed in Brazil vs 7.4% for studies performed in other countries; *P* = .50), the percentage of men (8.6% for studies with <50% men vs 2.3% for studies with >50% men; *P* = .27), or the route of disease transmission (4.4% for studies of vectorial transmission vs 5.1% for studies of oral transmission; *P* = .84) (eTable 1, eFigure 12, eFigure 13, and eFigure 14 in the Supplement). There were not enough individual data to perform subgroup analyses by age, type of study, or receipt of antitrypanosomal treatment. A model that included the percentage of men as a continuous variable estimated a rate increase of 5.6% per year (estimated rate ratio, 1.06; 95% CI, 0.87-1.33; *P* = .36; *I*^2^ = 80.1; τ^2^ [ln scale] = 0.6266) (eTable 1 and eFigure 15 in the Supplement).

A funnel plot for publication bias indicated missing studies in the middle and right side of the plot, suggesting that publication bias was plausible (eFigure 16 in the Supplement). Possible explanations for a noninterventional outcome measure included inadequate analysis, different effect sizes, sampling variation, and chance.^44^

## Discussion

We found a pooled estimated annual rate of 1.9% for the development of cardiomyopathy among patients with indeterminate chronic Chagas disease. This rate increased to 4.6% annually among patients diagnosed with acute Chagas infection. Among studies of patients with indeterminate chronic Chagas disease, we found an increased rate of cardiomyopathy development in studies that were conducted in Brazil, studies with shorter durations, and studies that did not include antiparasitic treatment. Researchers from Brazil have been pioneers in not only the discovery of Chagas disease but also in the performance of initial longitudinal studies of the disease. We hypothesize that earlier studies, which were mainly conducted in Brazil, had fewer treatment options available and were performed during a time when vectorial control measures were not as strict compared with more contemporary studies.

In studies in which patients received treatment with an antiparasitic drug, the progression to cardiomyopathy was significantly reduced compared with studies in which patients did not receive antiparasitic treatment. Longer observation periods in studies can introduce survival or disease-free biases. Male sex has also been associated with an increase in cardiomyopathy progression in several studies.^45,46^ Possible explanations include occupational differences that may be associated with an increased risk of continued exposure to vectors or intrinsic host-mediated factors. However, we could not assess the differences between sexes in our meta-analyses, probably owing to a lack of statistical power. Although we did not find an association between age and cardiomyopathy progression among patients with the indeterminate chronic form of Chagas disease, in many countries in which the disease is endemic, heart disease associated with Chagas infection is most frequently observed in older adults.

We also found an increase in the progression from acute infection to cardiomyopathy among studies conducted after 1975, likely because of the use and availability of more sensitive methods for the detection of cardiomyopathy. Most acute Chagas infections remain unnoticed because symptoms are mild, nonspecific, or even absent.^47^ Recognition of acute infection, although clinically challenging, becomes more important in endemic regions.

Our pooled estimates were consistent with those of previously published studies.^46,48^ One cohort study sponsored by the National Institutes of Health found an annual cardiomyopathy progression rate of 1.85% at 10 years of follow-up among patients with asymptomatic Chagas disease, which had been detected because the individuals had donated blood.^46^ A more recently published Brazilian cohort study also found a similar annual progression rate of 1.48% at 22 years of follow-up.^48^ Guidelines from the American Heart Association cite an annual rate of progression from the indeterminate chronic form of Chagas disease to cardiomyopathy of 1.85% to 7.0%.^49^

The process of cardiac injury during indeterminate chronic Chagas disease is multifactorial. The *T cruzi* genotype, persistent exposure to vectors, detection of parasitemia through polymerase chain reaction testing, oral acquisition, and recurrent infections have all been associated with cardiomyopathy onset and severity.^50,51,52^ The cumulative burden of disease observed in endemic areas suggest that persistent exposure to vectors is one of the main factors in the progression to cardiomyopathy. There is consensus that persistent tissue parasitism is associated with myocardial injury.^50^ Recurrent infections and substantial exposure to *T cruzi*, as observed with oral transmission, may be associated with more severe lesions.^51^

The higher risk of progression to cardiomyopathy that is observed in patients with acute Chagas disease suggests the need for rapid assessments of this population for the initiation of treatment to decrease disease morbidity. This higher risk also highlights the importance of ensuring that populations living in areas with high transmission rates have access to regular disease detection and treatment. This approach was instrumental in a *Trypanosoma brucei* elimination strategy that was implemented in Africa.^53^ However, only 1% to 2% (or less) of people with Chagas disease currently benefit from diagnosis and treatment.^54,55^

Oral transmission has also emerged as an important source of infection and warrants the implementation of public health measures to prevent contamination of food and drink. Annual cardiomyopathy progression rates of approximately 2% to 5% are substantial enough to explore the benefits of antitrypanosomal therapy at diagnosis (with either the indeterminate or acute form) coupled with strict vector control in endemic areas. Previous epidemiologic statements of indeterminate periods of 10 to 30 years may distract clinicians and public health authorities from the importance of exploring treatment options for patients with acute or indeterminate chronic forms of the disease. Annual estimates are a more accurate way to assess the short- and long-term risks of disease progression and provide real-time updated information to clinicians managing this condition when they are evaluating patients for treatment.

### Limitations

This study has several limitations. A substantial number of the observational studies included in our analysis had different epidemiologic settings and study designs, which translated to high heterogeneity. Given the relatively small samples, we could not be highly selective with the studies we used. The longitudinal studies on Chagas disease differed in their study populations, case selection processes, and follow-up durations.

The small samples were also a limitation for our subgroup analyses. Most studies used only changes in ECG results to determine the onset of chronic Chagas cardiomyopathy, which can decrease the sensitivity of cardiac injury detection and underestimate the risk of cardiomyopathy; however, the common use of ECG results added uniformity to the outcome’s definition. In contrast, the annual risk of cardiomyopathy development is typically estimated from the diagnosis of indeterminate chronic Chagas disease; however, patients may enter the latent phase of the disease several years before they receive a diagnosis.

The progression rate to cardiomyopathy is also nonlinear, as we assumed for this type of analysis. The rates of progression in patients with the early indeterminate form of Chagas disease are probably lower than those of patients with late indeterminate form. Few studies have included samples of younger patients with indeterminate chronic Chagas disease because most patients remain asymptomatic for 10 to 30 years after the initial infection (most often acquired during childhood through vector transmission), which is supported by the limited number of samples comprising younger patients with the indeterminate form that were included in this meta-analysis. Thus, the cardiomyopathy progression rate might be overestimated.

In this population, the mean follow-up duration was 8.5 years, with a maximum duration of 18 years. We were unable to identify whether the rate of cardiomyopathy development increased, remained stable, or decreased over time. We were also unable to extrapolate these data to patients with Chagas infection in nonendemic countries. It is possible that in nonendemic settings, which present little or no risk of reinfection, the progression to cardiomyopathy is delayed.

## Conclusions

To our knowledge, this systematic review addressed, for the first time in a scientific framework, the compiled data on factors associated with progression from the acute phase or the indeterminate chronic form of Chagas disease to the chronic cardiac form of the disease as well as the estimated annual rate of cardiomyopathy development. Based on our findings, a substantial cumulative risk of cardiomyopathy development may exist among those populations. Implementation and improvement of screening programs for Chagas disease that include assessment for antitrypanosomal treatment at the time of diagnosis are needed.

## Notes

Supplement. **eMethods.** Search Strategy, Selection Criteria, and Database Search Strategy **eTable 1.** Subgroup Analysis of Patients With the Indeterminate Chronic Form of Chagas Disease **eTable 2.** Subgroup Analysis of Patients With the Acute Form of Chagas Disease **eFigure 1.** Cumulative Risk of a Cardiac Event in Studies of Patients With the Indeterminate Chronic Form of Chagas Disease **eFigure 2.** Cumulative Risk of a Cardiac Event in Studies of Patients With the Acute Form of Chagas Disease **eFigure 3.** Subgroup Analysis by Year of Study for Studies of Patients With the Indeterminate Chronic Form of Chagas Disease **eFigure 4.** Subgroup Analysis by Study Size for Studies of Patients With the Indeterminate Chronic Form of Chagas Disease **eFigure 5.** Subgroup Analysis by Mean Age of Participants for Studies of Patients With the Indeterminate Chronic Form of Chagas Disease **eFigure 6.** Subgroup Analysis by Percentage of Men for Studies of Patients With the Indeterminate Chronic Form of Chagas Disease **eFigure 7.** Subgroup Analysis by Study’s Country of Origin for Studies of Patients With the Indeterminate Chronic Form of Chagas Disease **eFigure 8.** Subgroup Analysis by Use of Antitrypanosomal Treatment Intervention for Studies of Patients With the Indeterminate Chronic Form of Chagas Disease **eFigure 9.** Subgroup Analysis by Study Duration (in Years) for Studies of Patients With the Indeterminate Chronic Form of Chagas Disease **eFigure 10.** Forest Plot of Cardiomyopathy Risk in Studies of Patients With the Acute Form of Chagas Disease (Including the 6-Month Follow-Up Study) **eFigure 11.** Subgroup Analysis by Year of Study for Studies of Patients With the Acute Form of Chagas Disease **eFigure 12.** Subgroup Analysis by Study Size for Studies of Patients With the Acute Form of Chagas Disease **eFigure 13.** Subgroup Analysis by Study’s Country of Origin for Studies of Patients With the Acute Form of Chagas Disease **eFigure 14.** Subgroup Analysis by Route of Transmission for Studies of Patients With the Acute Form of Chagas Disease **eFigure 15.** Subgroup Analysis by Percentage of Men for Studies of Patients With the Acute Form of Chagas Disease **eFigure 16.** Funnel Plot for Publication Bias **eReferences**
