# Supplementary material for: Risk of Chronic Cardiomyopathy Among Patients With the Acute Phase or Indeterminate Form of Chagas Disease: A Systematic Review and Meta-analysis
Source: JAMA Netw Open. 2020 Aug 31;3(8):e2015072. doi: 10.1001/jamanetworkopen.2020.15072 (PMC7489816; doi:10.1001/jamanetworkopen.2020.15072)
Supplement: Supplement. — eMethods. Search Strategy, Selection Criteria, and Database Search Strategy eTable 1. Subgroup Analysis of Patients With the Indeterminate Chronic Form of Chagas Disease eTable 2. Subgroup Analysis of Patients With the Acute Form of Chagas Disease eFigure 1. Cumulative Risk of a Cardiac Event in Studies of Patients With the Indeterminate Chronic Form of Chagas Disease eFigure 2. Cumulative Risk of a Cardiac Event in Studies of Patients With the Acute Form of Chagas Disease eFigure 3. Subgroup Analysis by Year of Study for Studies of Patients With the Indeterminate Chronic Form of Chagas Disease eFigure 4. Subgroup Analysis by Study Size for Studies of Patients With the Indeterminate Chronic Form of Chagas Disease eFigure 5. Subgroup Analysis by Mean Age of Participants for Studies of Patients With the Indeterminate Chronic Form of Chagas Disease eFigure 6. Subgroup Analysis by Percentage of Men for Studies of Patients With the Indeterminate Chronic Form of Chagas Disease eFigure 7. Subgroup Analysis by Study’s Country of Origin for Studies of Patients With the Indeterminate Chronic Form of Chagas Disease eFigure 8. Subgroup Analysis by Use of Antitrypanosomal Treatment Intervention for Studies of Patients With the Indeterminate Chronic Form of Chagas Disease eFigure 9. Subgroup Analysis by Study Duration (in Years) for Studies of Patients With the Indeterminate Chronic Form of Chagas Disease eFigure 10. Forest Plot of Cardiomyopathy Risk in Studies of Patients With the Acute Form of Chagas Disease (Including the 6-Month Follow-Up Study) eFigure 11. Subgroup Analysis by Year of Study for Studies of Patients With the Acute Form of Chagas Disease eFigure 12. Subgroup Analysis by Study Size for Studies of Patients With the Acute Form of Chagas Disease eFigure 13. Subgroup Analysis by Study’s Country of Origin for Studies of Patients With the Acute Form of Chagas Disease eFigure 14. Subgroup Analysis by Route of Transmission for Studies of Patients With the Acute Form of [file jamanetwopen-e2015072-s001.pdf]

Supplementary Online Content

Chadalawada S, Sillau S, Archuleta S, et al. Risk of chronic cardiomyopathy among patients with the acute phase or indeterminate form of Chagas disease: a systematic review and meta-analysis. *JAMA Netw Open*. 2020;3(8):e2015072. doi:10.1001/jamanetworkopen.2020.15072

eMethods. Search Strategy, Selection Criteria, and Database Search Strategy

eTable 1. Subgroup Analysis of Patients With the Indeterminate Chronic Form of Chagas Disease

eTable 2. Subgroup Analysis of Patients With the Acute Form of Chagas Disease

eFigure 1. Cumulative Risk of a Cardiac Event in Studies of Patients With the Indeterminate Chronic Form of Chagas Disease

eFigure 2. Cumulative Risk of a Cardiac Event in Studies of Patients With the Acute Form of Chagas Disease

eFigure 3. Subgroup Analysis by Year of Study for Studies of Patients With the Indeterminate Chronic Form of Chagas Disease

eFigure 4. Subgroup Analysis by Study Size for Studies of Patients With the Indeterminate Chronic Form of Chagas Disease

eFigure 5. Subgroup Analysis by Mean Age of Participants for Studies of Patients With the Indeterminate Chronic Form of Chagas Disease

eFigure 6. Subgroup Analysis by Percentage of Men for Studies of Patients With the Indeterminate Chronic Form of Chagas Disease

eFigure 7. Subgroup Analysis by Study's Country of Origin for Studies of Patients With the Indeterminate Chronic Form of Chagas Disease

eFigure 8. Subgroup Analysis by Use of Antitrypanosomal Treatment Intervention for Studies of Patients With the Indeterminate Chronic Form of Chagas Disease

eFigure 9. Subgroup Analysis by Study Duration (in Years) for Studies of Patients With the Indeterminate Chronic Form of Chagas Disease

eFigure 10. Forest Plot of Cardiomyopathy Risk in Studies of Patients With the Acute Form of Chagas Disease (Including the 6-Month Follow-Up Study)

eFigure 11. Subgroup Analysis by Year of Study for Studies of Patients With the Acute Form of Chagas Disease

eFigure 12. Subgroup Analysis by Study Size for Studies of Patients With the Acute Form of Chagas Disease

eFigure 13. Subgroup Analysis by Study's Country of Origin for Studies of Patients With the Acute Form of Chagas Disease

© 2020 Chadalawada S et al. *JAMA Network Open*.

eFigure 14. Subgroup Analysis by Route of Transmission for Studies of Patients With the Acute Form of Chagas Disease

eFigure 15. Subgroup Analysis by Percentage of Men for Studies of Patients With the Acute Form of Chagas Disease

eFigure 16. Funnel Plot for Publication Bias

eReferences

This supplementary material has been provided by the authors to give readers additional information about their work.

© 2020 Chadalawada S et al. *JAMA Network Open*.

eMethods. Search Strategy, Selection Criteria, and Database Search Strategy

Search strategy and selection criteria:

This review considered longitudinal studies, prospective and retrospective cohort studies, randomized and non-randomized clinical trials, case-control studies, and time-series studies. Descriptive cross-sectional studies were also included depending on the outcome's measurements. Case reports were excluded. Indeterminate Chagas disease must be confirmed through positive serologic testing for Chagas disease and the absence of structural cardiomyopathy with no cardiac symptoms, and a normal ECG. Studies also must include a longitudinal observation of participants with the acute phase of the infection or already established indeterminate form of the disease until the development of a primary outcome (i.e. cardiomyopathy manifestations). Studies were excluded if they did not state enough or pertinent outcome data or were determined not to have an acceptable quality methodologic assessment. Keywords including Chagas disease, development of cardiomyopathy, latency duration and determinants of the Chagas indeterminate period were included in the search. A comprehensive literature search was performed by a medical librarian from October 8, 2018 to October 24, 2018. Relevant publications were identified by searching a combination of indexing terms (when applicable, specific to each database) and keywords for the concepts of Chagas disease progression to cardiomyopathy. The following databases were searched: MEDLINE (via Ovid MEDLINE® and Epub Ahead of Print, In-Process & Other Non-Indexed Citations, Daily and Versions®, 1946 to present), Web of Science Core Collection (via Clarivate Analytics, including Science Citation Index Expanded 1974 to present, and Social Sciences Citation Index 1974 to present), Embase (via Elsevier, Embase.com, 1947 to present), Cochrane Library (via Wiley, including Cochrane Database of Systematic Reviews and Cochrane Central Register of Controlled Trials), and LILACS (Latin American and Caribbean Health Sciences Literature, via BV Salud, 1982 to present). A search of MEDLINE (via PubMed, 1946 to present) was conducted on February 20, 2019 to identify any new publications since the original search. The reference list of all studies selected for critical appraisal was screened for additional studies, and other important articles in the field were manually added. There were no restrictions on the date of publication or age of subjects. Publication language was restricted to English, Spanish or Portuguese. Articles written in Spanish or Portuguese were reviewed by the authors (AHM, CFP, AR) who are fluent in both languages. Filters were used to limit results to human subjects. After the search, all identified studies were uploaded and de-duplicated in EndNote VX8 (Clarivate Analytics, PA, USA). Covidence systematic review software (Veritas Health Innovation, Melbourne, Australia) was used for screening and full text review. Through Covidence, a Preferred Reporting Items for Systematic Reviews and Meta-Analyses (PRISMA) flow diagram was generated with the number of results found, number excluded during title/abstract screening, and number excluded during full-text assessments and methodological appraisals, along with reasons for exclusion. Other sources searched included ClinicalTrials.gov (United States National Library of Medicine) and the World Health Organization International Clinical Trials Registry Platform (ICTRP, <http://www.who.int/ictrp/en/>). Two potentially relevant studies were identified in ClinicalTrials.gov, but in both cases, the authors had published their findings in papers that were already identified in the Ovid MEDLINE search. We sought summary estimates data.

Database search strategy

MEDLINE (via Ovid MEDLINE(R) and Epub Ahead of Print, In-Process & Other Non-Indexed Citations, Daily and Versions(R) 1946 to present)
Search date = 10/22/2018

1. exp Chagas Disease/
2. exp Trypanosoma cruzi/
3. Chagas.tw,kf.
4. Chagasic.tw,kf.

© 2020 Chadalawada S et al. *JAMA Network Open*.

5. Trypanosoma cruzi.tw,kf.
6. American trypanosomiasis.tw,kf.
7. OR/1-6
8. exp Disease Progression/
9. latent.tw,kf.
10. latency.tw,kf.
11. indeterminate.tw,kf.
12. asymptomatic.tw,kf.
13. exp longitudinal studies/
14. longitudinal stud*.tw,kf.
15. exp cohort studies/
16. cohort stud*.tw,kf.
17. exp follow-up studies/
18. follow up stud*.tw,kf.
19. exp prospective studies/
20. prospective stud*.tw,kf.
21. exp case-control studies/
22. case control stud*.tw,kf.
23. observational stud*.tw,kf.
24. progression.tw,kf.
25. progressive.tw,kf.
26. evolution.tw,kf.
27. OR/8-26
28. 7 AND 27
29. exp animals/ NOT exp humans/
30. 28 NOT 29

MEDLINE (via PubMed, 1946 to present)
Search date = 2/20/2019
1. "Chagas Disease"[mesh]
2. "Trypanosoma cruzi"[mesh]
3. Chagas[tw]
4. Chagasic[tw]
5. "Trypanosoma cruzi"[tw]
6. "American trypanosomiasis"[tw]
7. #1 OR #2 OR #3 OR #4 OR #5 OR #6
8. "Disease Progression"[mesh]
9. latent[tw]
10. latency[tw]
11. indeterminate[tw]
12. asymptomatic[tw]
13. "longitudinal studies"[mesh]
14. longitudinal stud*[tw]
15. "cohort studies"[mesh]
16. cohort stud*[tw]
17. "follow-up studies"[mesh]
18. follow-up stud*[tw]
19. "prospective studies"[mesh]
20. prospective stud*[tw]
21. "case-control studies"[mesh]
22. case-control stud*[tw]

© 2020 Chadalawada S et al. *JAMA Network Open*.

23. observational stud*[tw]
24. progression[tw]
25. progressive[tw]
26. evolution[tw]
27. #8 OR #9 OR #10 OR #11 OR #12 OR #13 OR #14 OR #15 OR #16 OR #17 OR #18 OR #19 OR #20 OR #21 OR #22 OR #23 OR #24 OR #25 OR #26
28. #7 AND #27
29. "animals"[mesh] NOT "humans"[mesh]
30. #28 NOT #29

Web of Science Core Collection (via Thomson Reuters, including Science Citation Index Expanded 1974 to present, and Social Sciences Citation Index 1974 to present)

Search date = 10/22/2018

1. TS=("Chagas")
2. TS=("Chagasic")
3. TS=("Trypanosoma cruzi")
4. TS=("American trypanosomiasis")
5. #1 OR #2 OR #3 OR #4
6. TS=("latent")
7. TS=("latency")
8. TS=("indeterminate")
9. TS=("asymptomatic")
10. TS=("longitudinal stud*")
11. TS=("cohort stud*")
12. TS=("follow up stud*")
13. TS=("prospective stud*")
14. TS=("case control stud*")
15. TS=("observational stud*")
16. TS=("progression")
17. TS=("progressive")
18. TS=("evolution")
19. #6 OR #7 OR #8 OR #9 OR #10 OR #11 OR #12 OR #13 OR #14 OR #15 OR #16 OR #17 OR #18
20. #5 AND #19

Embase (via Elsevier, Embase.com, 1947 to present)

Search date = 10/22/2018

1. 'Chagas disease'/exp
2. 'Trypanosoma cruzi'/exp
3. Chagas:ab,ti
4. Chagasic:ab,ti
5. Trypanosoma cruzi':ab,ti
6. American trypanosomiasis':ab,ti
7. #1 OR #2 OR #3 OR #4 OR #5 OR #6
8. 'latent period'/exp
9. 'disease exacerbation'/exp
10. 'longitudinal study'/exp
11. 'cohort analysis'/exp
12. 'follow up'/exp
13. 'prospective study'/exp
14. 'case control study'/exp
15. latent:ab,ti

© 2020 Chadalawada S et al. *JAMA Network Open*.

16. latency:ab,ti
17. indeterminate:ab,ti
18. asymptomatic:ab,ti
19. longitudinal stud*':ab,ti
20. cohort stud*':ab,ti
21. follow up stud*':ab,ti
22. prospective stud*':ab,ti
23. case control stud*':ab,ti
24. observational stud*':ab,ti
25. progression:ab,ti
26. progressive:ab,ti
27. evolution:ab,ti
28. #8 OR #9 OR #10 OR #11 OR #12 OR #13 OR #14 OR #15 OR #16 OR #17 OR #18 OR #19 OR #20 OR #21 OR #22 OR #23 OR #24 OR #25 OR #26 OR #27
29. #7 AND #28
30. [animals]/lim NOT [humans]/lim
31. #29 NOT #30

Cochrane Library (via Wiley, including Cochrane Database of Systematic Reviews and Cochrane Central Register of Controlled Trials)

Search date = 10/22/2018

1. MeSH descriptor: [Chagas Disease] explode all trees
2. MeSH descriptor: [Trypanosoma cruzi] explode all trees
3. (chagas):ti,ab,kw OR (chagasic):ti,ab,kw OR ("Trypanosoma cruzi"):ti,ab,kw OR ("American trypanosomiasis"):ti,ab,kw
4. #1 OR #2 OR #3
5. MeSH descriptor: [Disease Progression] explode all trees
6. MeSH descriptor: [Longitudinal Studies] explode all trees
7. MeSH descriptor: [Cohort Studies] explode all trees
8. MeSH descriptor: [Follow-Up Studies] explode all trees
9. MeSH descriptor: [Prospective Studies] explode all trees
10. MeSH descriptor: [Case-Control Studies] explode all trees
11. (latent):ti,ab,kw OR (latency):ti,ab,kw OR (indeterminate):ti,ab,kw OR (asymptomatic):ti,ab,kw OR (longitudinal NEXT stud*):ti,ab,kw
12. (cohort NEXT stud*):ti,ab,kw OR (follow NEXT up NEXT stud*):ti,ab,kw OR (prospective NEXT stud*):ti,ab,kw OR (case NEXT control NEXT stud*):ti,ab,kw OR (observational NEXT stud*):ti,ab,kw
13. (progression):ti,ab,kw OR (progressive):ti,ab,kw OR (evolution):ti,ab,kw
14. #5 OR #6 OR #7 OR #8 OR #9 OR #10 OR #11 OR #12 OR #13
15. #4 AND #14

LILACS (Latin American and Caribbean Health Sciences Literature, via BV Salud, 1982 to October 2018)

Search date = 10/24/2018

tw:((chagas OR chagasic OR "Trypanosoma cruzi" OR "American trypanosomiasis") AND (asintomatic* OR asymptomatic OR "case control study" OR "caso control" OR cohort OR "estudio longitudinal" OR "estudo longitudinal" OR evolucao OR evolucion OR evolution OR "follow up study" OR inalterada OR indeterminad* OR indeterminate OR latencia OR latency OR latent OR "longitudinal study" OR observacoes OR "observational study" OR progresion OR progressao OR progression OR progressive OR prospectiv* OR "prospective study" OR seguimiento)) AND (instance:"regional") AND (db:("LILACS") AND type:("article"))

© 2020 Chadalawada S et al. *JAMA Network Open*.

tw:((chagas OR chagasic OR "Trypanosoma cruzi" OR "American trypanosomiasis") AND (asintomatic* OR asymptomatic OR "case control study" OR "caso control" OR cohort OR "estudio longitudinal" OR "estudo longitudinal" OR evolucao OR evolucion OR evolution OR "follow up study" OR inalterada OR indeterminad* OR indeterminate OR latencia OR latency OR latent OR "longitudinal study" OR observacoes OR "observational study" OR progresion OR progressao OR progression OR progressive OR prospectiv* OR "prospective study" OR seguimiento)) AND (instance:"regional") AND (db:("LILACS") AND type:("article"))

Summary

**Database**	**# Results**
Ovid Medline	2187
PubMed	2306
Web of Science	2252
Embase	2809
Cochrane	74
LILACS	1133
Subtotal	10761
Duplicates removed	5756
**Total unique results**	**5005**

© 2020 Chadalawada S et al. *JAMA Network Open*.

eTable 1. Subgroup Analysis of Patients With the Indeterminate Chronic Form of Chagas Disease[1-23]

Subgroups	Estimated Annual Rate (%)	95% CI	I^2	*p-values*
**Year study**				0.102
After 1985	1.4	0.8-2.2	86.8%	
Before 1985	2.8	1.7-4.8	98.3%	
**Study size**				0.828
>200	2.1	1.0-4.2	99.2%	
<200	1.9	1.1-3.2	90.0%	
**Country**				0.047
Other	1.1	0.5-2.4	95.0%	
Brazil	2.3	1.2-4.3	97.6%	
**Age**				0.838
> 32	1.6	0.8-2.9	94.7%	
< 32	2.4	0.8-7.8	93.5%	
**Men %**				0.455
< 40%	1.0	0.4-2.6	81.5%	
> 40%	2.2	0.9-5.1	99.0%	
**Antiparasitic Intervention**				0.028
No	2.3	1.5-3.5	97.9%	
Yes	1.0	0.5-1.9	71.9%	
**Study duration**				0.001
< 9 years	3.2	2.0-5.0	98.5%	
≥ 9 years	1.2	0.7-2.1	95.3%	

© 2020 Chadalawada S et al. *JAMA Network Open*.

eTable 2. Subgroup Analysis of Patients With the Acute Form of Chagas Disease[24-32]

Subgroups	Estimated Annual Rate (%)	95% CI	I^2	*p-values*
**Year of study onset**				0.007
After 1975	10.1	4.9-21	54.6%	
Before 1975	2.9	1.6-5.4	87.7%	
**Study size**				0.421
>40	3.8	2.1-6.8	87.5%	
<40	7.2	1.6-32.3	88.9%	
**Country**				0.498
Other	7.4	1.5-36.8	87.2%	
Brazil	4.0	2.2-7.4	88.1%	
**Percentage of men**				0.271
< 50%	8.6	2.5-29.3	84.0%	
> 50%	2.3	1.2-4.5	23.6%	
**Type of transmission**				0.836
Vector	4.4	2-9.5	88.5%	
Oral	5.1	2-13.3	83.5%	

© 2020 Chadalawada S et al. *JAMA Network Open*.

eFigure 1. Cumulative Risk of a Cardiac Event in Studies of Patients With the Indeterminate Chronic Form of Chagas Disease

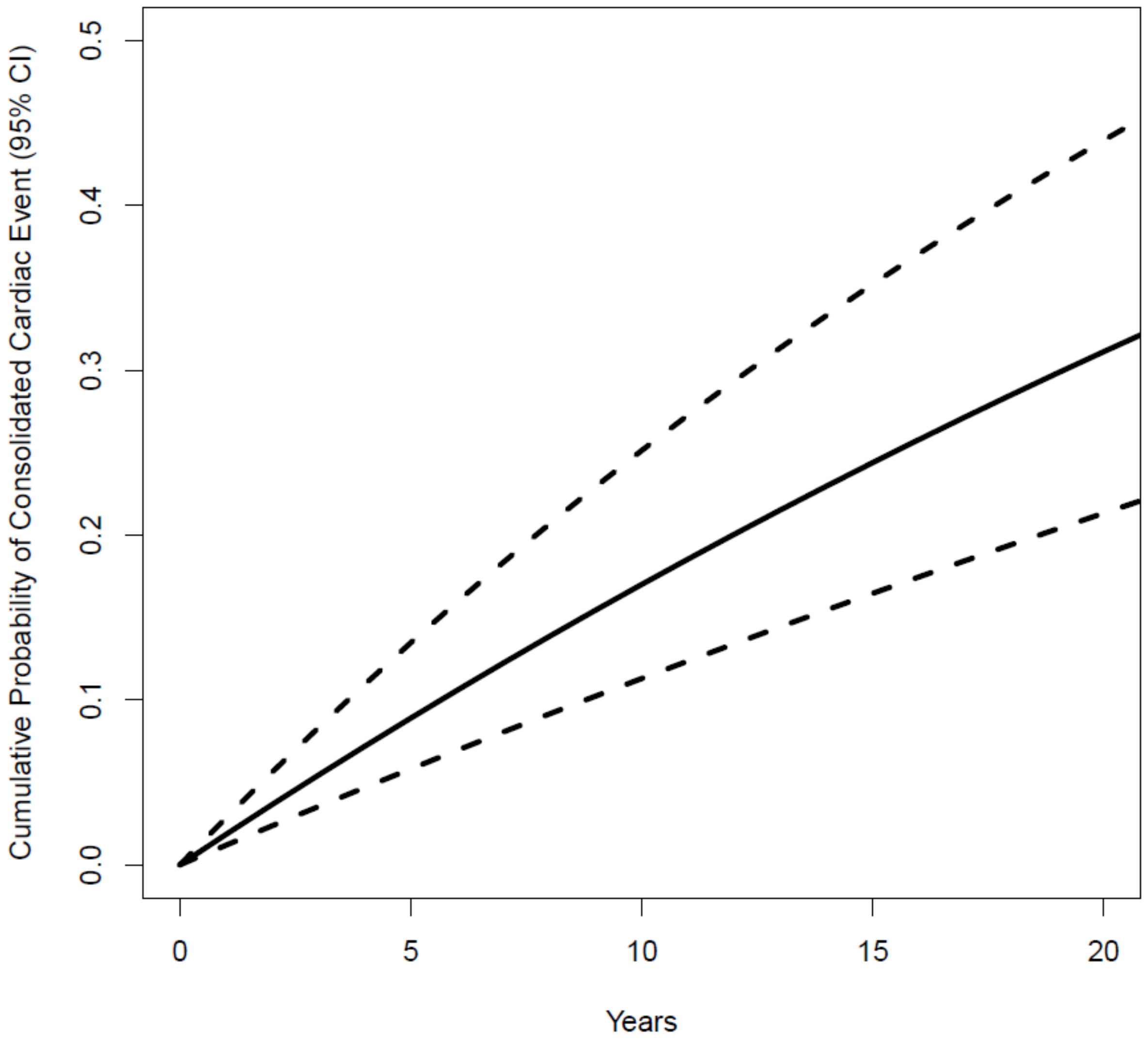

© 2020 Chadalawada S et al. *JAMA Network Open*.

eFigure 2. Cumulative Risk of a Cardiac Event in Studies of Patients With the Acute Form of Chagas Disease

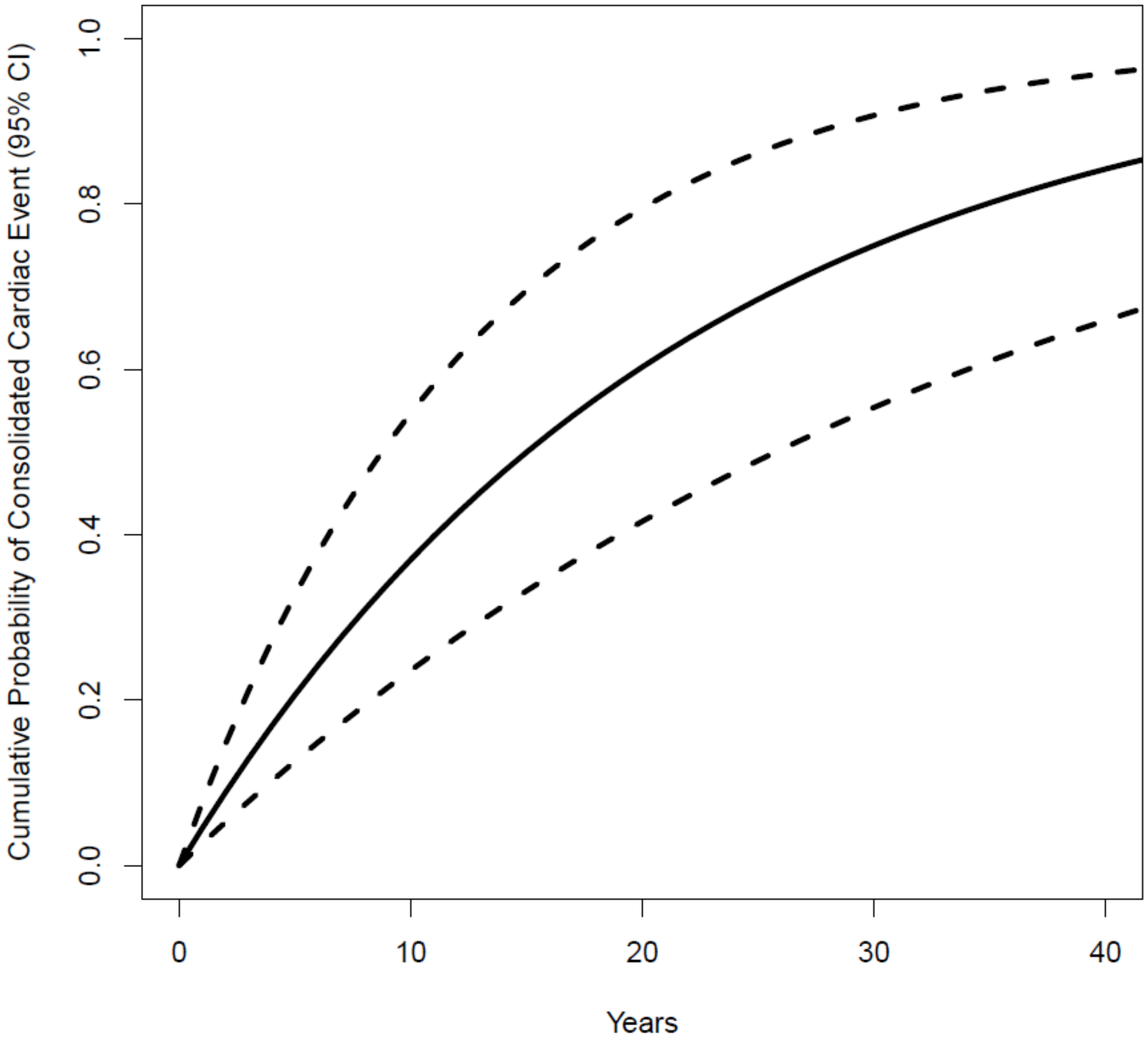

© 2020 Chadalawada S et al. *JAMA Network Open*.

eFigure 3. Subgroup Analysis by Year of Study for Studies of Patients With the Indeterminate Chronic Form of Chagas Disease

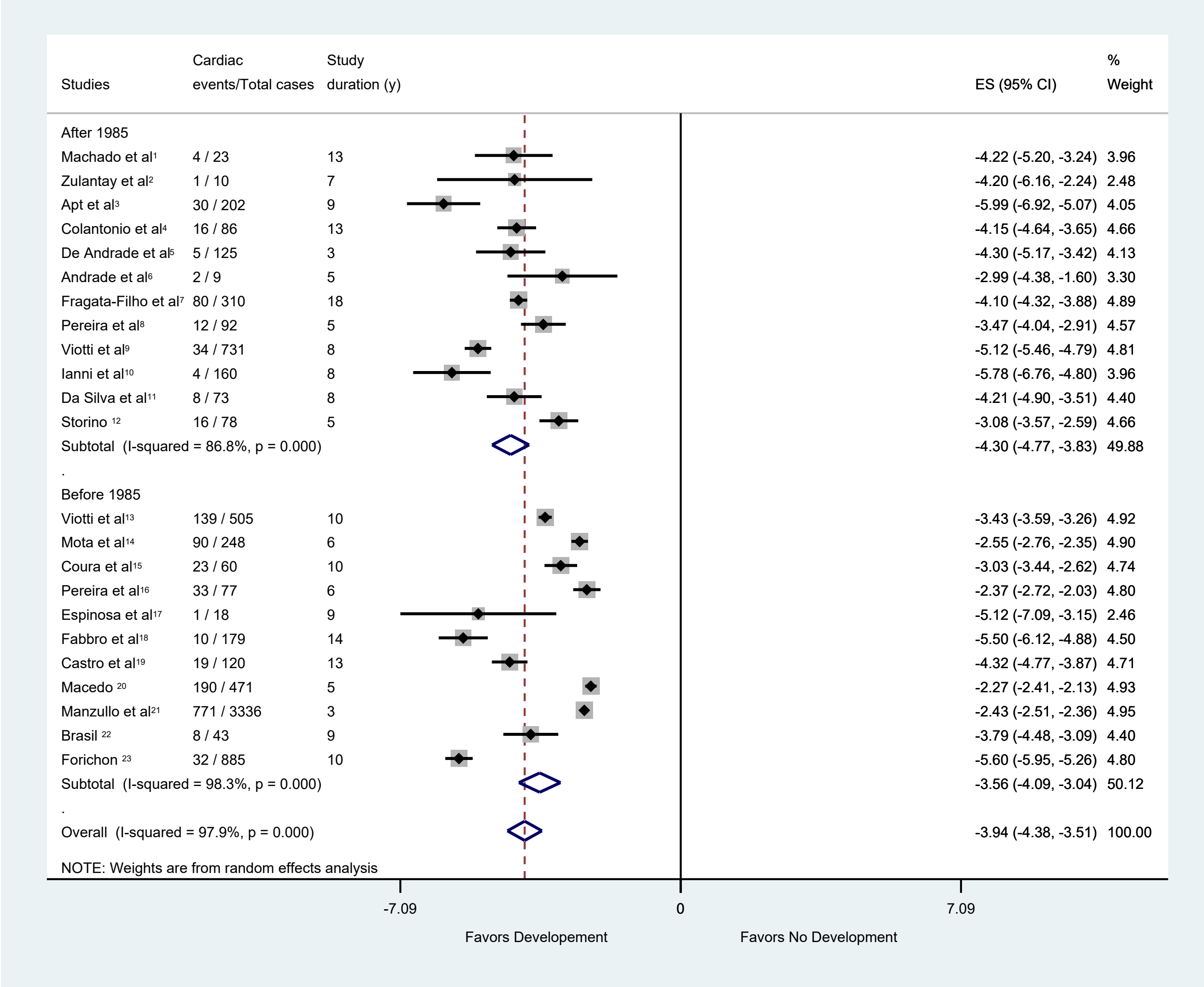

Greater negative logarithmic rate estimates convert to a lower back transformed rate

© 2020 Chadalawada S et al. *JAMA Network Open*.

eFigure 4. Subgroup Analysis by Study Size for Studies of Patients With the Indeterminate Chronic Form of Chagas Disease

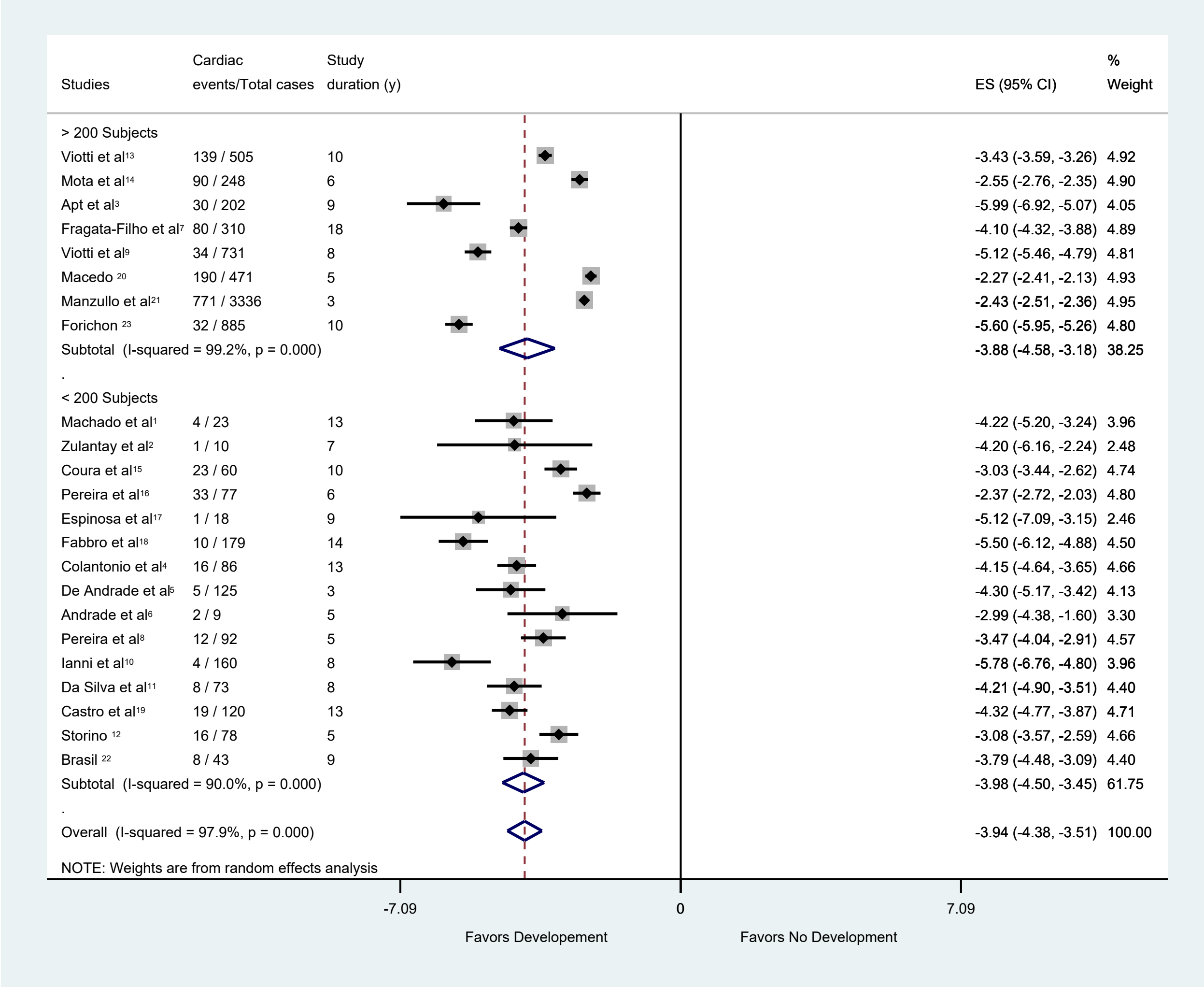

Greater negative logarithmic rate estimates convert to a lower back transformed rate

© 2020 Chadalawada S et al. *JAMA Network Open*.

eFigure 5. Subgroup Analysis by Mean Age of Participants for Studies of Patients With the Indeterminate Chronic Form of Chagas Disease

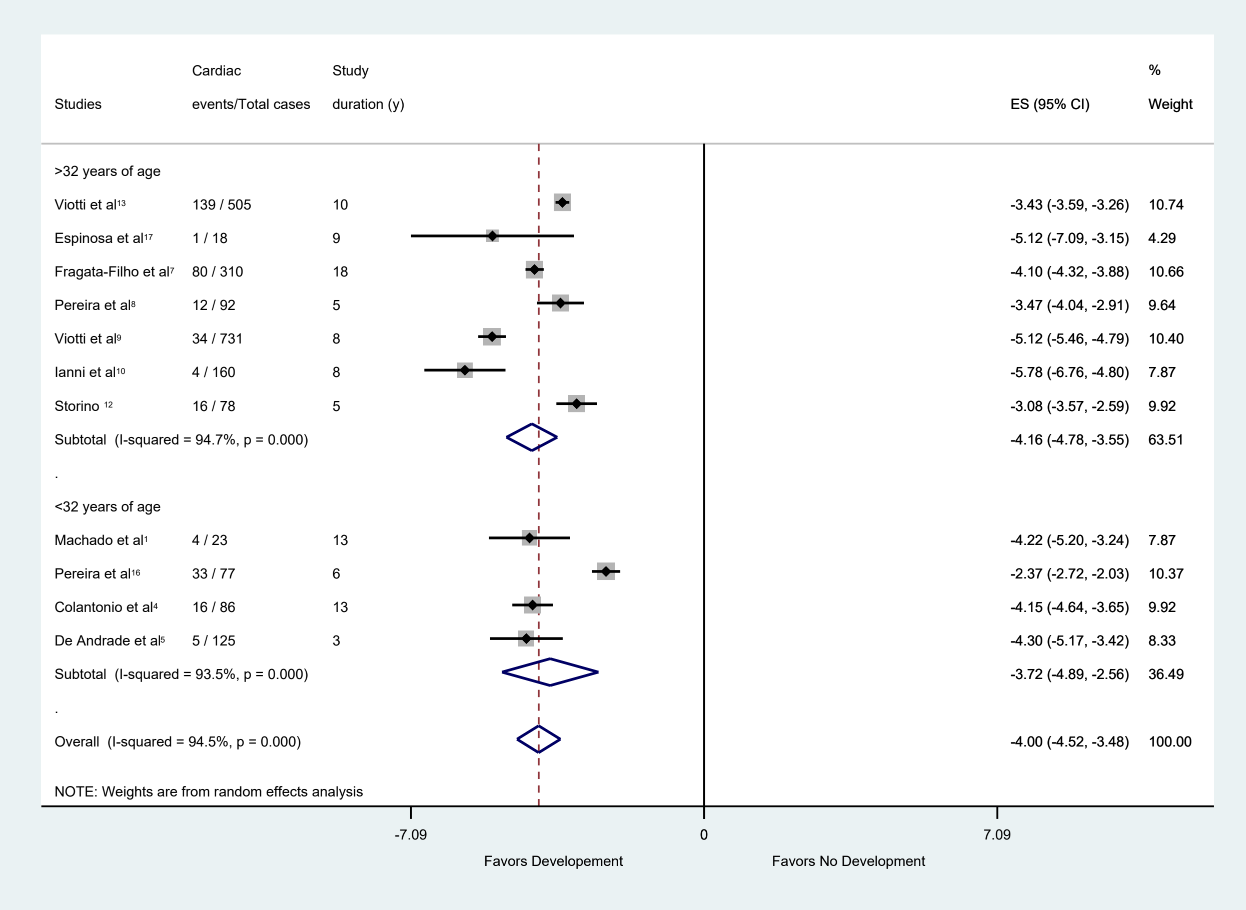

Greater negative logarithmic rate estimates convert to a lower back transformed rate

© 2020 Chadalawada S et al. *JAMA Network Open*.

eFigure 6. Subgroup Analysis by Percentage of Men for Studies of Patients With the Indeterminate Chronic Form of Chagas Disease

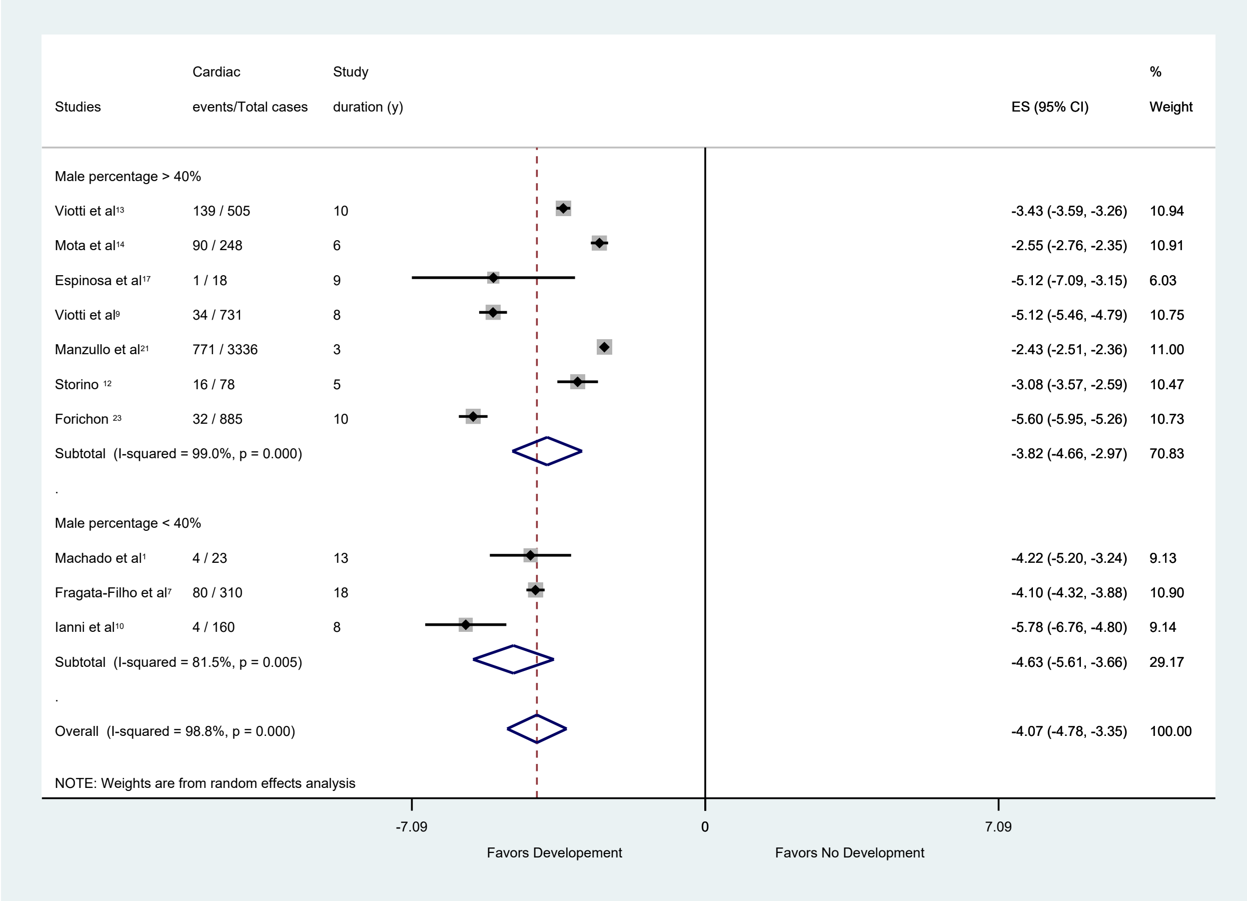

Greater negative logarithmic rate estimates convert to a lower back transformed rate

© 2020 Chadalawada S et al. *JAMA Network Open*.

eFigure 7. Subgroup Analysis by Study’s Country of Origin for Studies of Patients With the Indeterminate Chronic Form of Chagas Disease

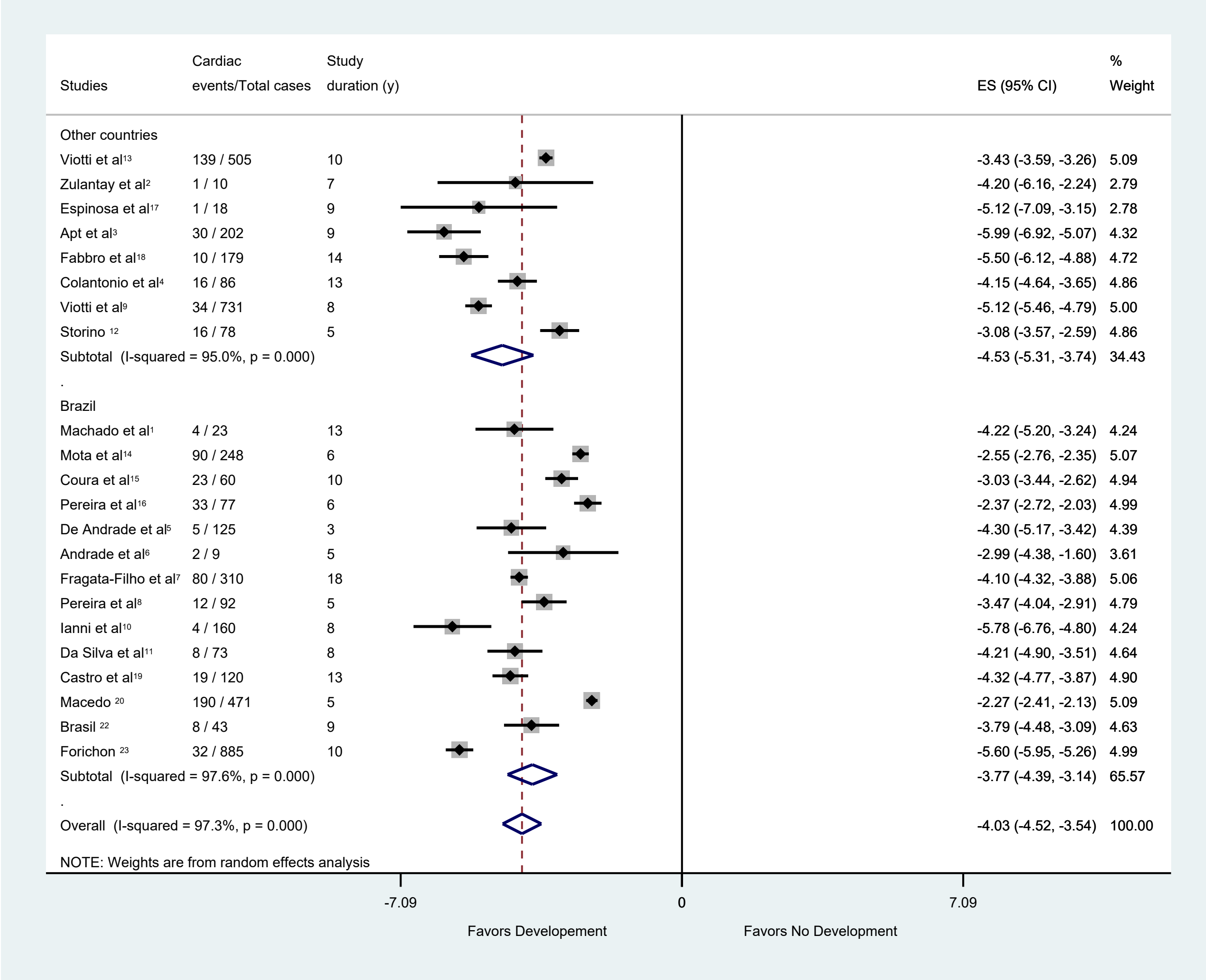

Greater negative logarithmic rate estimates convert to a lower back transformed rate

© 2020 Chadalawada S et al. *JAMA Network Open*.

eFigure 8. Subgroup Analysis by Use of Antitrypanosomal Treatment Intervention for Studies of Patients With the Indeterminate Chronic Form of Chagas Disease

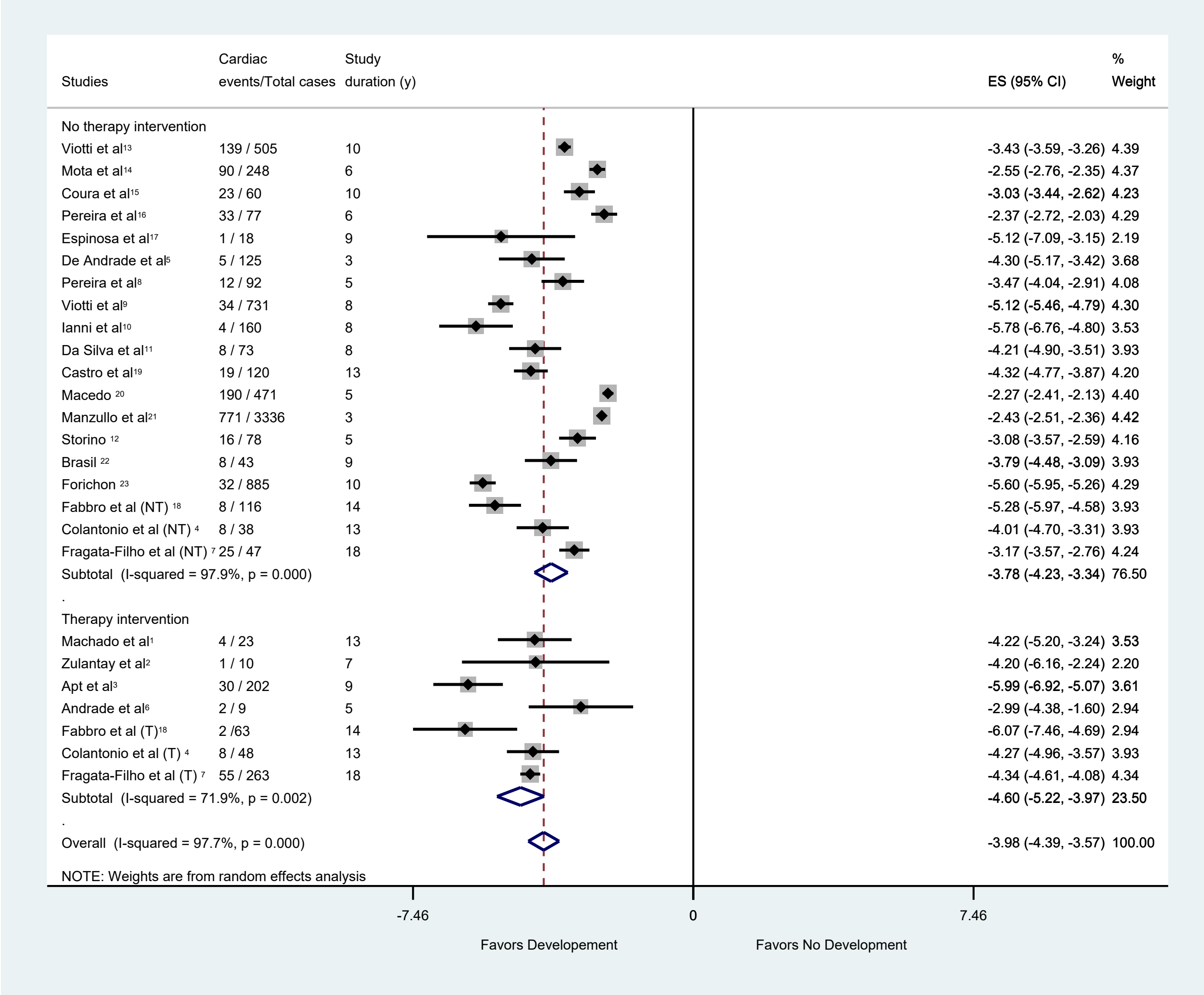

Greater negative logarithmic rate estimates convert to a lower back transformed rate

© 2020 Chadalawada S et al. *JAMA Network Open*.

eFigure 9. Subgroup Analysis by Study Duration (in Years) for Studies of Patients With the Indeterminate Chronic Form of Chagas Disease

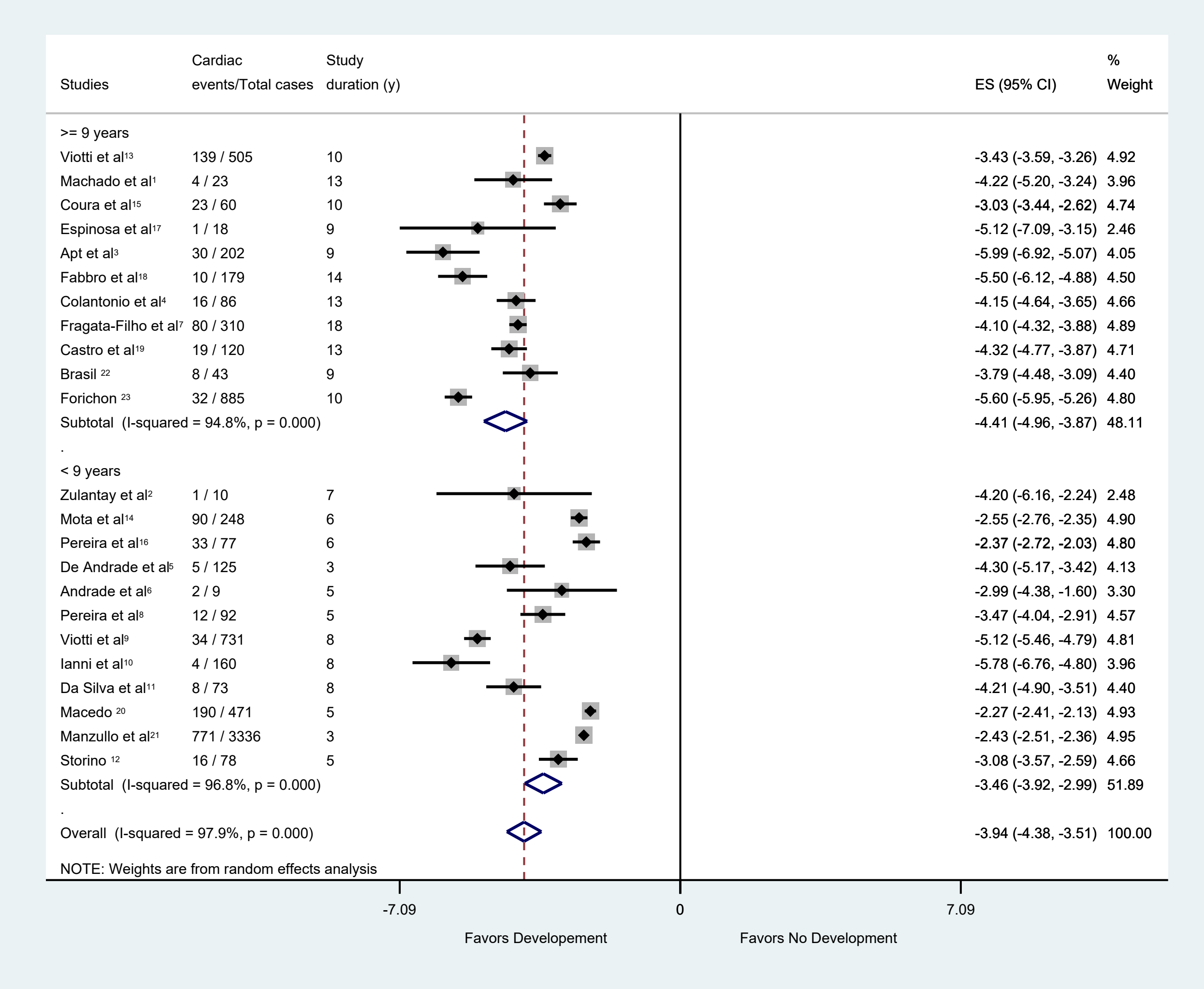

Greater negative logarithmic rate estimates convert to a lower back transformed rate

© 2020 Chadalawada S et al. *JAMA Network Open*.

eFigure 10. Forest Plot of Cardiomyopathy Risk in Studies of Patients With the Acute Form of Chagas Disease (Including the 6-Month Follow-Up Study)

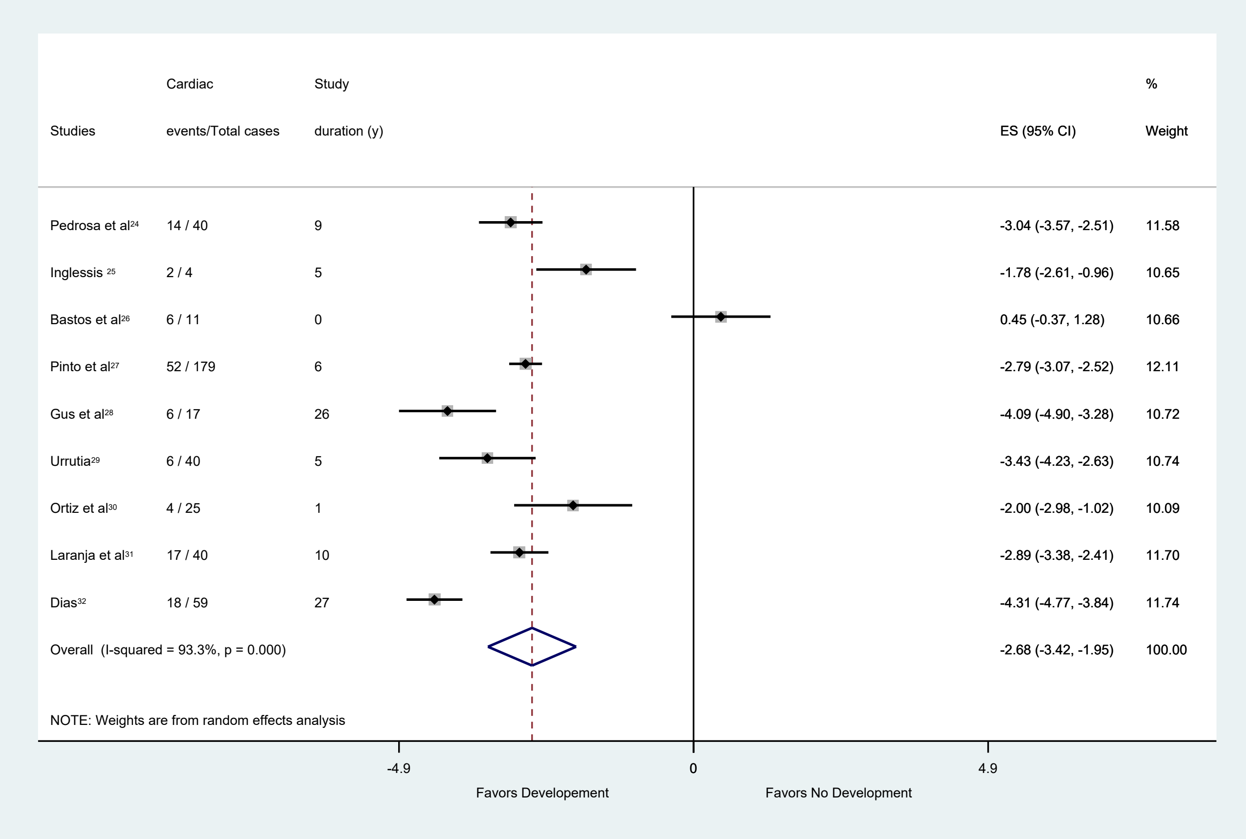

Greater negative logarithmic rate estimates convert to a lower back transformed rate

© 2020 Chadalawada S et al. *JAMA Network Open*.

eFigure 11. Subgroup Analysis by Year of Study for Studies of Patients With the Acute Form of Chagas Disease

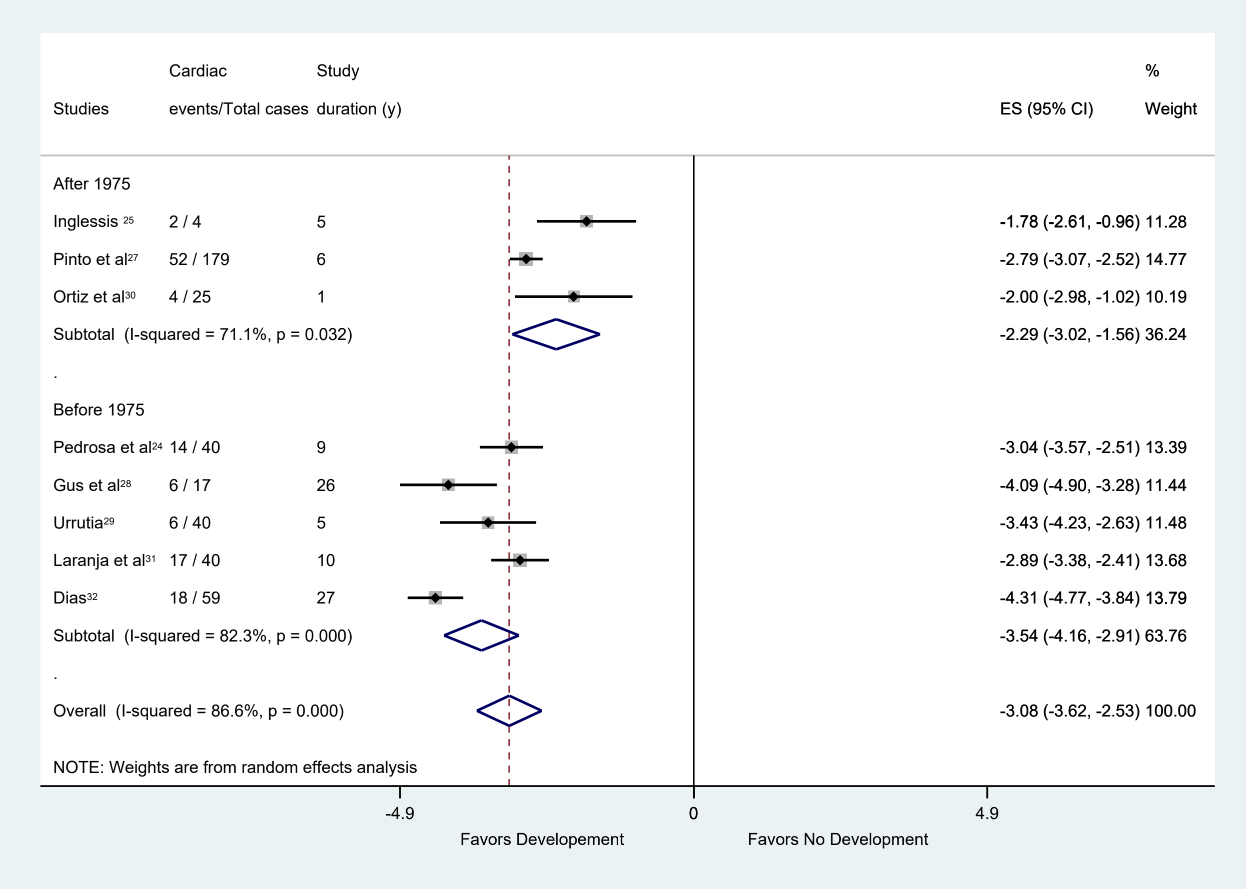

Greater negative logarithmic rate estimates convert to a lower back transformed rate

© 2020 Chadalawada S et al. *JAMA Network Open*.

eFigure 12. Subgroup Analysis by Study Size for Studies of Patients With the Acute Form of Chagas Disease

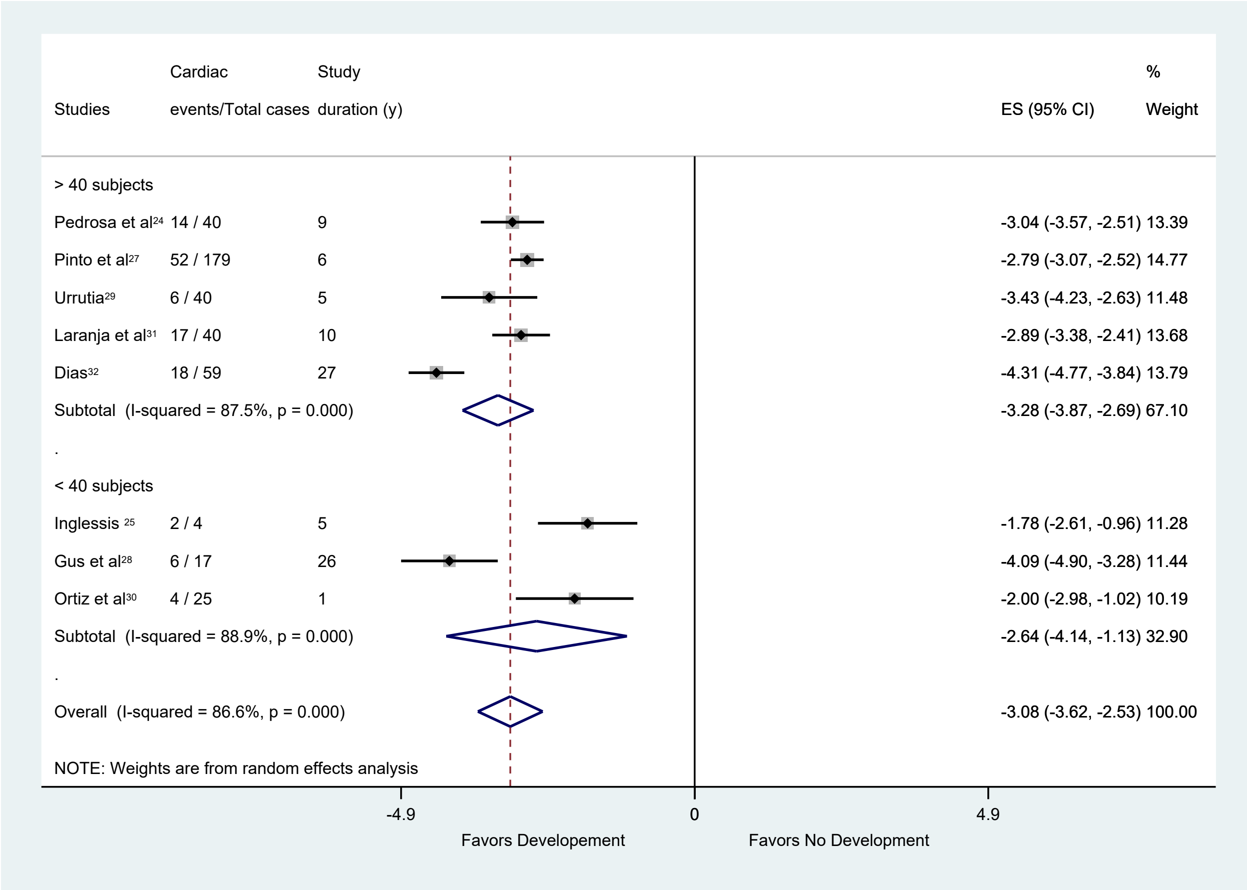

Greater negative logarithmic rate estimates convert to a lower back transformed rate

© 2020 Chadalawada S et al. *JAMA Network Open*.

eFigure 13. Subgroup Analysis by Study’s Country of Origin for Studies of Patients With the Acute Form of Chagas Disease

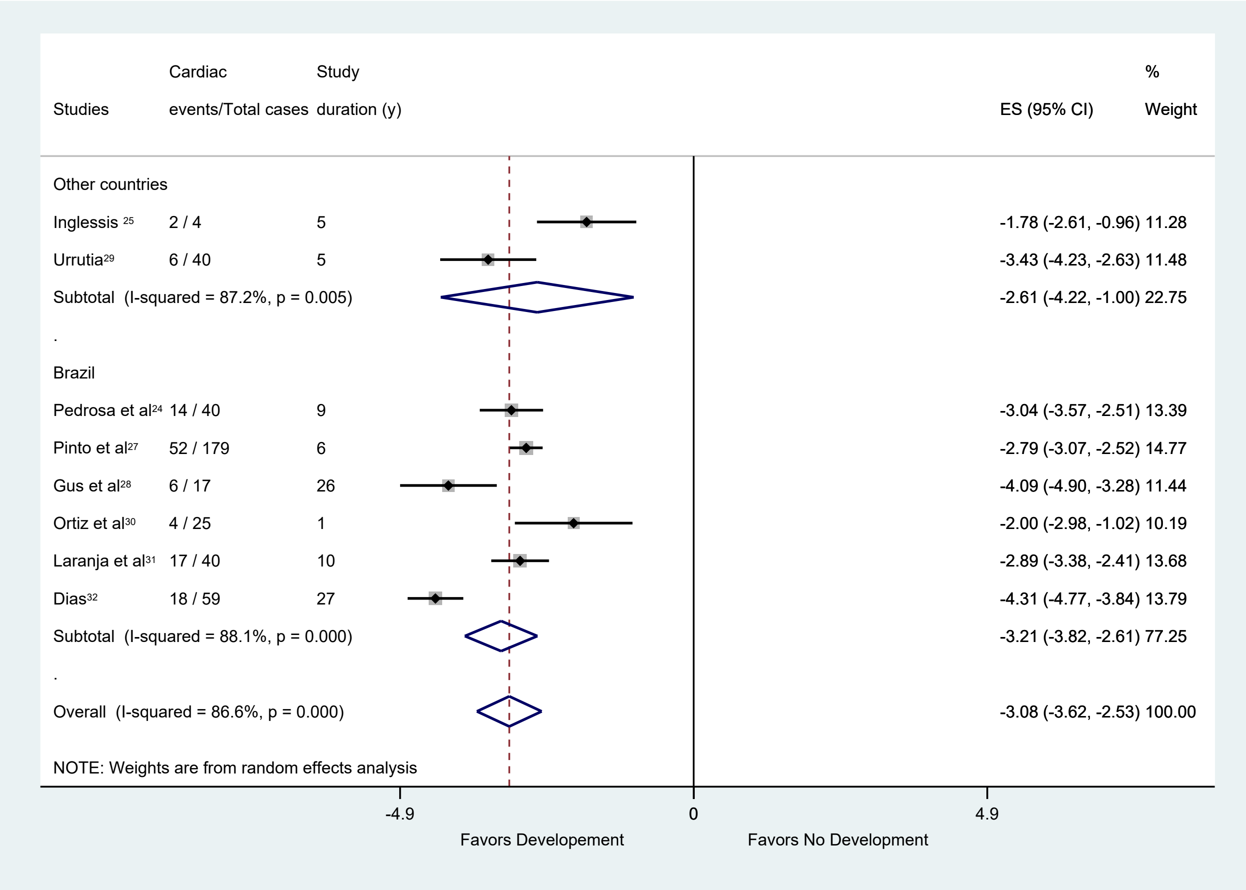

Greater negative logarithmic rate estimates convert to a lower back transformed rate

© 2020 Chadalawada S et al. *JAMA Network Open*.

eFigure 14. Subgroup Analysis by Route of Transmission for Studies of Patients With the Acute Form of Chagas Disease

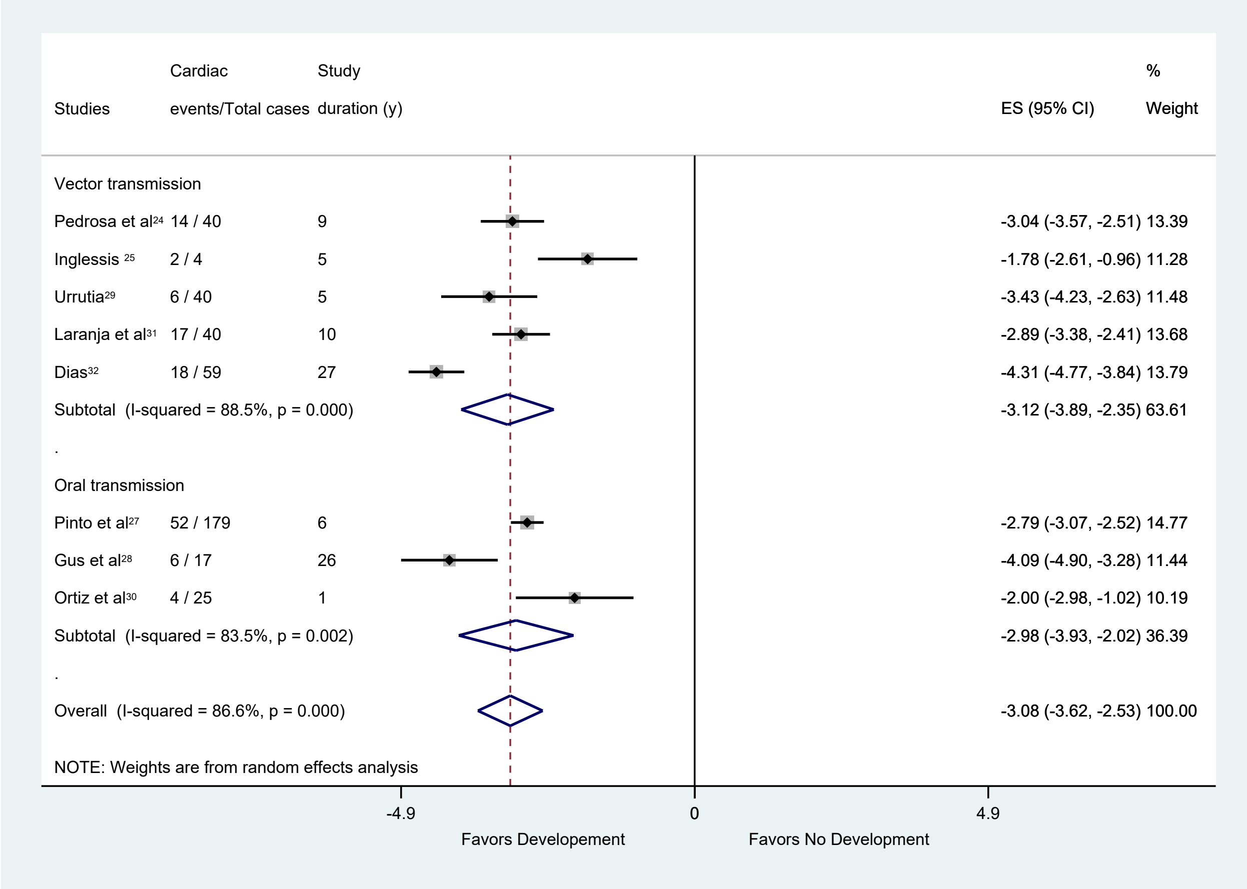

Greater negative logarithmic rate estimates convert to a lower back transformed rate

© 2020 Chadalawada S et al. *JAMA Network Open*.

eFigure 15. Subgroup Analysis by Percentage of Men for Studies of Patients With the Acute Form of Chagas Disease

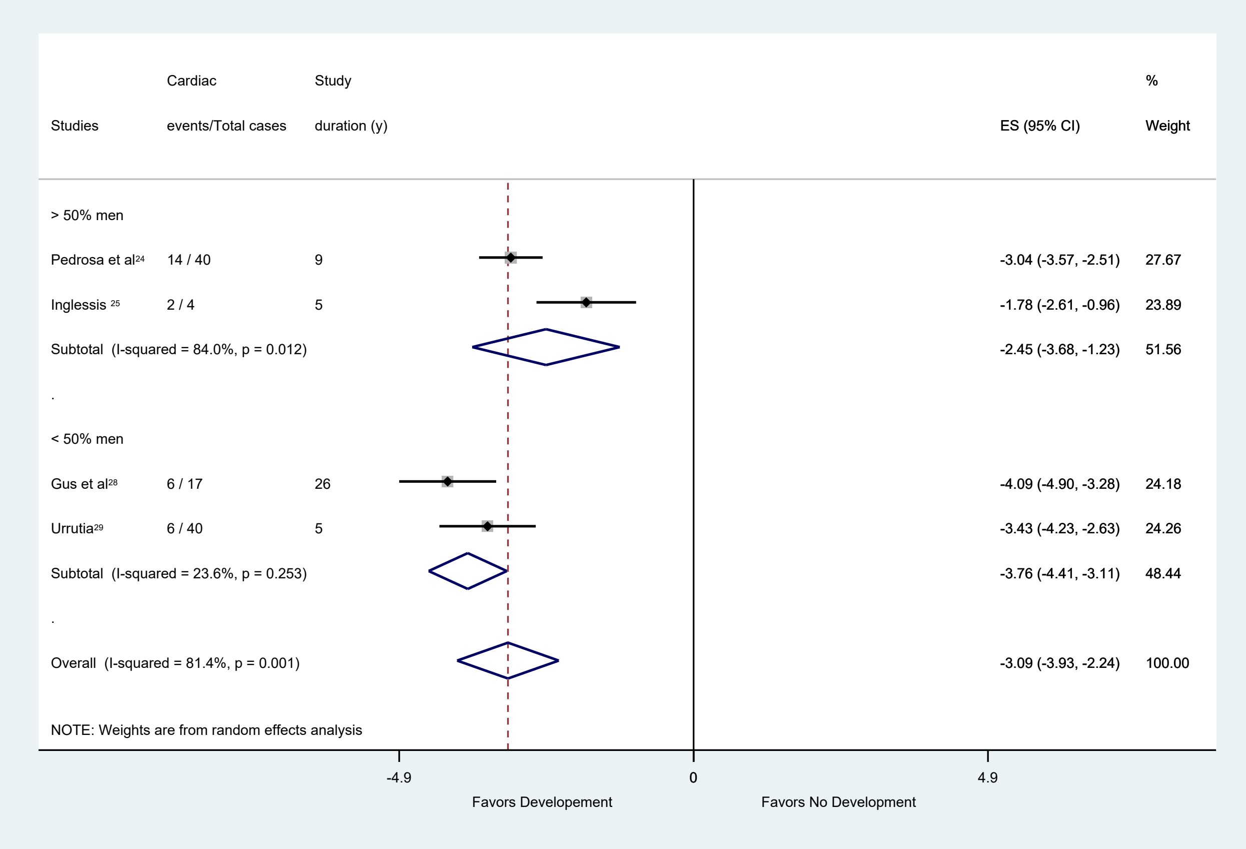

Greater negative logarithmic rate estimates convert to a lower back transformed rate

© 2020 Chadalawada S et al. *JAMA Network Open*.

eFigure 16. Funnel Plot for Publication Bias

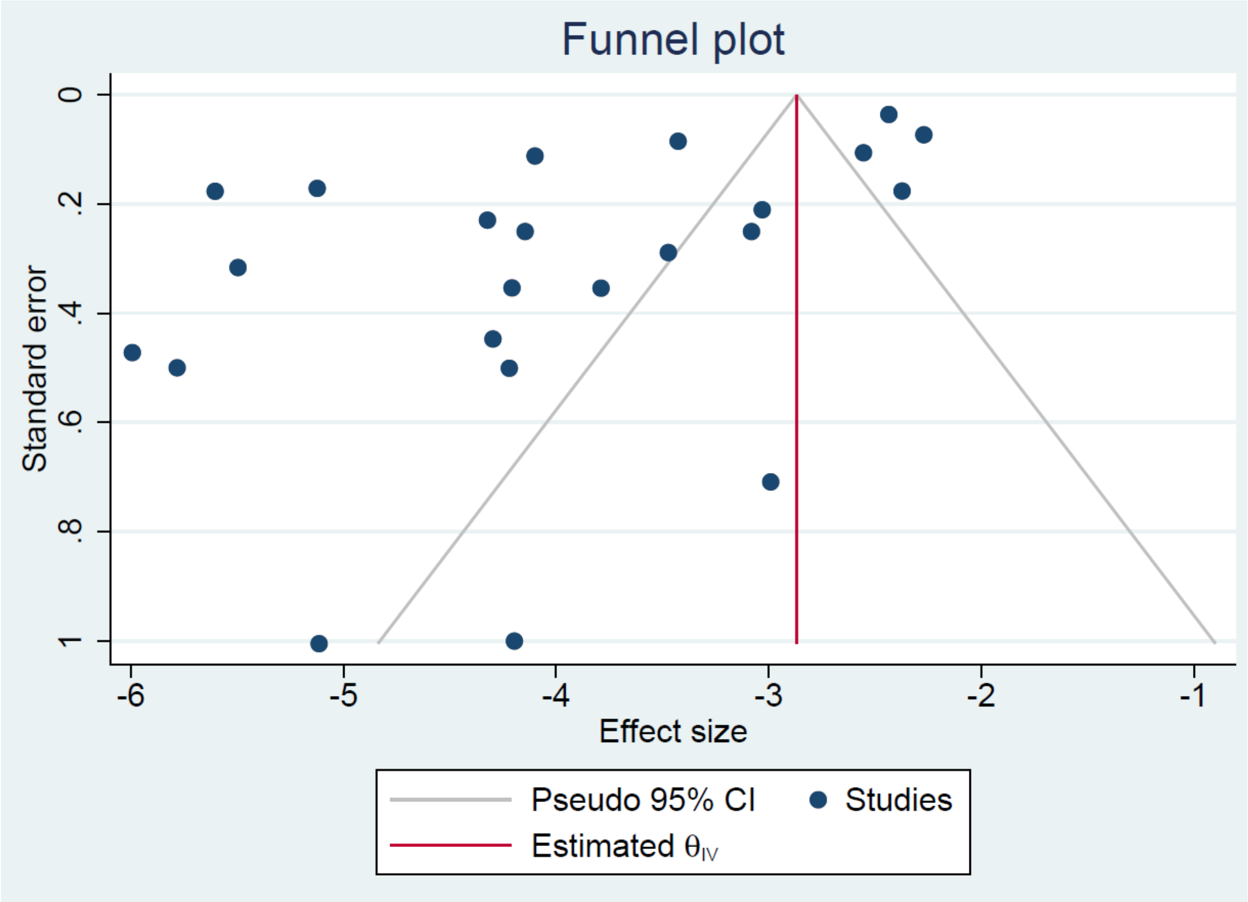

© 2020 Chadalawada S et al. *JAMA Network Open*.

eReferences

1. Machado-de-Assis GF, Diniz GA, Montoya RA, et al. A serological, parasitological and clinical evaluation of untreated Chagas disease patients and those treated with benznidazole before and thirteen years after intervention. *Memorias do Instituto Oswaldo Cruz.* 2013;108(7):873-880.
2. Zulantay I, Arribada A, Honores P, et al. [No association between persistence of the parasite and electrocardiographic evolution in treated patients with Chagas disease]. *Revista Medica de Chile.* 2005;133(10):1153-1160.
3. Apt W, Arribada A, Zulantay I, Sanchez G, Vargas SL, Rodriguez J. Itraconazole or allopurinol in the treatment of chronic American trypanosomiasis: The regression and prevention of electrocardiographic abnormalities during 9 years of follow-up. *Annals of tropical medicine and parasitology.* 2003;97(1):23-29.
4. Colantonio LD, Prado N, Segura EL, Sosa-Estani S. Electrocardiographic Abnormalities and Treatment with Benznidazole among Children with Chronic Infection by Trypanosoma cruzi: A Retrospective Cohort Study. *PLoS Neglected Tropical Diseases [electronic resource].* 2016;10(5):e0004651.
5. de Andrade AL, Zicker F, Rassi A, et al. Early electrocardiographic abnormalities in Trypanosoma cruzi-seropositive children. *American Journal of Tropical Medicine & Hygiene.* 1998;59(4):530-534.
6. Andrade MC, Rocha EA, Costa ACd, et al. Evolução eletrocardiográfica e sorológica de pacientes com doença de Chagas crônica acompanhados por seis anos após o tratamento com benzonidazol. *RELAMPA, Rev Lat-Am Marcapasso Arritm.* 2016;29(4):f:141-l:149.
7. Fragata-Filho AA, Franca FF, Fragata Cda S, Lourenco AM, Faccini CC, Costa CA. Evaluation of Parasiticide Treatment with Benznidazol in the Electrocardiographic, Clinical, and Serological Evolution of Chagas Disease. *PLoS Neglected Tropical Diseases [electronic resource].* 2016;10(3):e0004508.

© 2020 Chadalawada S et al. *JAMA Network Open*.

8. Pereira JB, da Cunha RV, Willcox HP, Coura JR. [Development of chronic human chagas cardiopathy in the hinterland of the Paraiba State, Brazil, in a 4.5 year period]. *Revista Da Sociedade Brasileira de Medicina Tropical.* 1990;23(3):141-147.
9. Viotti R, Vigliano C, Lococo B, et al. [Clinical predictors of chronic chagasic myocarditis progression]. *Revista Espanola de Cardiologia.* 2005;58(9):1037-1044.
10. Ianni BM, Mady C, Arteaga E, Fernandes F. [Cardiovascular diseases observed during follow-up of a group of patients with undetermined form of Chagas' disease]. *Arquivos Brasileiros de Cardiologia.* 1998;71(1):21-24.
11. da Silva MA, Costa JM, Barbosa JM, et al. [Chronic phase of Chagas disease. Clinical aspects and course of the disease]. *Arquivos Brasileiros de Cardiologia.* 1994;63(4):281-285.
12. Storino R MJ. Estudios de seguimiento evolutivo de la enfermedad de Chagas. In: *Madoery RJ, Madoery C, Cámera MI (eds). Actualizaciones en la enfermedad de Chagas.* Córdoba, Argentina1993:67-77.
13. Viotti RJ, Vigliano C, Laucella S, et al. Value of echocardiography for diagnosis and prognosis of chronic Chagas disease cardiomyopathy without heart failure. *Heart.* 2004;90(6):655-660.
14. Mota EA, Guimaraes AC, Santana OO, Sherlock I, Hoff R, Weller TH. A nine year prospective study of Chagas' disease in a defined rural population in northeast Brazil. *American Journal of Tropical Medicine & Hygiene.* 1990;42(5):429-440.
15. Coura JR, Abreu LLd, Pereira JB, Willcox HP. Morbidity in Chagas' disease: IV. Longitudinal study of 10 years in Pains and Iguatama, Minas Gerais, Brazil. *Memórias do Instituto Oswaldo Cruz.* 1985;80(1):73-80.
16. Pereira JB, Willcox HP, Coura JR. [Morbidity in Chagas' disease. III. Longitudinal study of 6 years, in Virgem da Lapa, MG, Brazil]. *Memorias do Instituto Oswaldo Cruz.* 1985;80(1):63-71.
17. Espinosa R, Carrasco HA, Belandria F, et al. Life expectancy analysis in patients with Chagas' disease: prognosis after one decade (1973-1983). *International Journal of Cardiology.* 1985;8(1):45-56.

© 2020 Chadalawada S et al. *JAMA Network Open*.

18. Fabbro De Suasnabar D, Arias E, Streiger M, et al. Evolutive behavior towards cardiomyopathy of treated (nifurtimox or benznidazole) and untreated chronic chagasic patients. *Revista do Instituto de Medicina Tropical de Sao Paulo.* 2000;42(2):99-109.

19. Castro C, Prata A, Macedo V. [A 13-year clinical study on 190 chronic chagasic patients from Mambai, Goias, Brazil]. *Revista Da Sociedade Brasileira de Medicina Tropical.* 2001;34(4):309-318.

20. Macedo V. Influência da exposição à reinfecção na evolução da doença de Chagas. Estudo longitudinal de cinco anos. *Revista PatologiaTropical.* 1976;5:33-116.

21. Manzullo ECD, Miguel A; Libonatti, Osvaldo; Rozlosnik, Jorge. *ESTUDIO LONGITUDINAL DE LA CARDIOPATIA CHAGASICA CRONICA*. http://www.enfermedadchagas.com.ar/Librocompleto.pdf: CENTRO DE CHAGAS DE LA CATEDRA DE ENFERMEDADES INFECCIOSAS DE LA FACULTAD DE CIENCIAS MEDICAS DE BUENOS AIRES, Universidad de Buenos Aires; 1982.

22. Brasil A. Evolucao e prognostico da doenca de Chagas. *Arq Bra Cardio.* 1965;18:365-380.

23. Forichon E. Contribution aux estimations de morbidite et de mortalite dans la maladie de Chagas. *Rev Pat Trop.* 1975;4(1):57-78.

24. Pedrosa RC, Cancado JR, Decache W. [A longitudinal electrocardiogram study of Chagas' disease from the acute phase]. *Revista Da Sociedade Brasileira de Medicina Tropical.* 1993;26(3):163-174.

25. Inglessis I, Carrasco HA, Anez N, et al. [Clinical, parasitological and histopathologic follow-up studies of acute Chagas patients treated with benznidazole]. *Archivos del Instituto de Cardiologia de Mexico.* 1998;68(5):405-410.

26. Bastos CJ, Aras R, Mota G, et al. Clinical outcomes of thirteen patients with acute chagas disease acquired through oral transmission from two urban outbreaks in northeastern Brazil. *PLoS Neglected Tropical Diseases [electronic resource].* 2010;4(6):e711.

© 2020 Chadalawada S et al. *JAMA Network Open*.

27. Pinto AY, Valente Vda C, Coura JR, et al. Clinical follow-up of responses to treatment with benznidazol in Amazon: a cohort study of acute Chagas disease. *PLoS ONE [Electronic Resource]*. 2013;8(5):e64450.

28. Gus I, Molon ME, Bueno AP. [Chagas disease--review of 8 simultaneous cases of acute Chagas myocarditis: 25 years later]. *Arquivos Brasileiros de Cardiologia.* 1993;60(2):99-101.

29. Urrutia LE. Acute chagasic myocardiopathy in children. Five years evolution. *Archivos del Instituto de Cardiologia de Mexico.* 1976;46(4):396-413.

30. Ortiz JV, Pereira BVM, Couceiro KDN, et al. Cardiac Evaluation in the Acute Phase of Chagas' Disease with Post-Treatment Evolution in Patients Attended in the State of Amazonas, Brazil. *Arquivos Brasileiros de Cardiologia.* 2019.

31. Laranja FS, Dias E, Miranda A, Nobrega G. Chagas' disease; a clinical, epidemiologic, and pathologic study. *Circulation.* 1956;14(6):1035-1060.

32. Dias JC, Dias E, Nobrega GC. Long-term follow-up of a patient since the acute phase of Chagas disease (South American trypanosomiasis): further treatment and cure of the infection. *Revista Da Sociedade Brasileira de Medicina Tropical.* 2015;48(5):629-632.
